# Non-cancer mortality among firefighters: a meta-analytic review of heart disease, stroke, respiratory disease, liver disease, accidents, and suicide

**DOI:** 10.3389/fpubh.2026.1714033

**Published:** 2026-02-26

**Authors:** Paulo S. Pinheiro, Soyeon Ahn, Stephanie Negron, Victoria Pinilla, Erin N. Kobetz, Alberto J. Caban-Martinez, David J. Lee

**Affiliations:** 1 Department of Public Health Sciences, Leonard M. Miller School of Medicine, University of Miami, Miami, FL, United States; 2 Sylvester Comprehensive Cancer Center, Leonard M. Miller School of Medicine, University of Miami, Miami, FL, United States; 3 Department of Education and Psychological Studies, University of Miami, Miami, FL, United States; 4 Department of Medicine, Leonard M. Miller School of Medicine, University of Miami, Miami, FL, United States; 5 Florida Cancer Data System, Florida Department of Health, Miami, FL, United States

**Keywords:** accidents, cause of death, firefighters, heart disease, liver disease, mortality, respiratory disease, stroke

## Abstract

**Introduction:**

Firefighting is a hazardous occupation linked to elevated cancer risk. However, occupational exposures unique to this profession may also contribute to non-cancer morbidity and mortality. To better understand firefighters’ health risks, it is essential to examine causes of death beyond cancer.

**Methods:**

Following PRISMA guidelines, we conducted a systematic review and meta-analysis of population-based studies published between 1978 and 2025. Studies reporting standardized mortality ratios (SMRs) for non-cancer causes of death were identified through database searches. We estimated pooled SMRs and 95% confidence intervals using random-effects models, tested for publication bias and study quality, and conducted moderator analyses.

**Results:**

Twenty-five studies were included for final meta-analysis. Firefighters exhibited significantly lower mortality rate for heart disease (SMR = 0.64; 95%CI: 0.51–0.80), cerebrovascular disease (SMR = 0.67; 95%CI: 0.50–0.90), diabetes mellitus (SMR = 0.48; 95%CI: 0.32–0.70), intentional self-harm/suicide (SMR = 0.52; 95%CI: 0.39–0.70), chronic lower respiratory disease (SMR = 0.71; 95%CI: 0.55–0.91), and accidents/injuries (SMR = 0.77; 95%CI: 0.62–0.98) compared to the general population. In contrast, evidence for respiratory infections (*k* = 3) and liver disease (*k* = 6) was sparse and mortality estimates were comparable to those in the general population. Meta-regression analyses revealed no significant difference across studied moderators including study location, occupational data sources, incident types attended, gender, race, employment status, smoking status, or study quality score on the observed mortality patterns.

**Conclusion:**

Firefighters experience lower mortality from multiple non-cancer causes, potentially due to occupational fitness requirements and the healthy worker effect. However, parity in mortality from liver disease and pneumonia/influenza warrant further investigation into behavioral factors and occupational exposures. Our results refine the healthy worker effect by identifying cause-specific gaps and priorities for targeted prevention and surveillance.

## 1 Introduction

Firefighting is a physically demanding and high-risk profession that exposes workers to hazardous work conditions, harmful chemical substances, and life-threatening emergencies. In the United States (U.S.), over one million individuals serve as firefighters (1), facing significant occupational health risks. Recognizing these dangers, the International Agency for Research on Cancer (IARC) has classified firefighting-related occupational exposures as carcinogenic to humans (Group 1) (2). Epidemiological studies have linked firefighting to increased risks for multiple cancers, including multiple myeloma, non-Hodgkin lymphoma, prostate, bladder and testicular cancers (2–5).

Beyond cancer, firefighters also face significant morbidity and mortality risks from other causes, some directly linked to their occupational hazards. Exposure to smoke, combustion byproducts, and structural collapse debris may contribute to an increased risk of cardiovascular and respiratory diseases. According to the U.S. Fire Administration, nearly 40% of on-duty firefighter deaths in 2022 were attributed to cardiovascular and cerebrovascular events, followed by 18% from motor vehicle trauma, with other causes, including falls, structural collapses, and environmental exposures, accounting for the remaining fatalities (6). When considering both on-duty and off-duty firefighters, and excluding malignancies, the leading causes of death include cardiovascular, respiratory, and digestive diseases, as well as injuries related to violence (including suicide) (7).

While previous meta-analytical studies have primarily focused on cancer-related mortality and morbidity, a notable gap remains in the literature regarding non-cancer causes of death among firefighters (4). Understanding these causes is crucial, as the same occupational exposures linked to cancer may also increase mortality from cardiovascular, respiratory, or liver diseases. To our knowledge, no meta-analysis has systematically examined non-cancer mortality in this workforce.

In this study, we address this gap by conducting a comprehensive meta-analysis of non-cancer causes of death among firefighters. We systematically review and synthesize existing studies on mortality due to heart disease, unintentional injuries (accidents), cerebrovascular diseases, chronic lower respiratory diseases, chronic liver disease and cirrhosis, suicide, diabetes mellitus, and pneumonia/influenza. These causes were selected because they represent leading contributors to firefighter mortality, are plausibly linked to occupational exposures or psychosocial stressors inherent to firefighting, and are commonly considered in occupational injury, disability, and death-benefit determinations (8–12). By carefully selecting studies with minimal geographical and chronological overlap and conducting moderator analyses, we aim to identify key sources of variation and provide more precise estimates of cause-specific mortality patterns among firefighters. This work contributes to a more comprehensive understanding of non-cancer mortality among firefighters, beyond the well-documented cancer burden.

## 2 Methods

### 2.1 Search strategy

Relevant studies published between January 1978 and December 2025 were identified through a series of comprehensive electronic searches of multiple databases including PUBMED, EMBASE, Web of Science, SCOPUS, and ProQuest, as well as ERIC, PsycINFO and Medline via EBSCO. To ensure relevance and consistency, we applied specific filters to limit the search results to articles published in English, studies involving human subjects, and populations aged 19 years and older. Keywords being used in all our searches are a combination of the following terms: (Mortality OR cause of death OR “cause specific mortality”) & (fire inspector OR fire inspectors OR fire rescue OR fire-rescue OR firefighter OR firefighters OR “fire fighter” OR “fire fighters” OR paramedic OR paramedics OR emergency medical technician OR “first responder”). We included the final four terms in our search strategy to account for departments that provide both fire and rescue services. Articles identified using these terms were reviewed to determine whether firefighting was among the occupational responsibilities of the workers described. Studies in which firefighting was not a component of the job duties were excluded from the meta-analysis. The detailed search strategies, including MeSH terms, used for each database are provided in the Supplementary material. Two independent reviewers were responsible for determining if studies were eligible with a third author confirming any discrepancies in whether a study met eligibility criteria.

#### 2.1.1 Inclusion and exclusion criteria

To be included in the current meta-analysis, a study must meet the following inclusion and exclusion criteria, including:

1. A study must be empirical and quantitative;
2. A study must be based on human research;
3. A study population must be about firefighters;
4. Any study must include at least one non-cancer related cause of death among firefighters;
5. A study must be written in English;
6. A study must report sufficient statistics to enable the computation of an effect size and its associated standard error.

**Figure 1:**
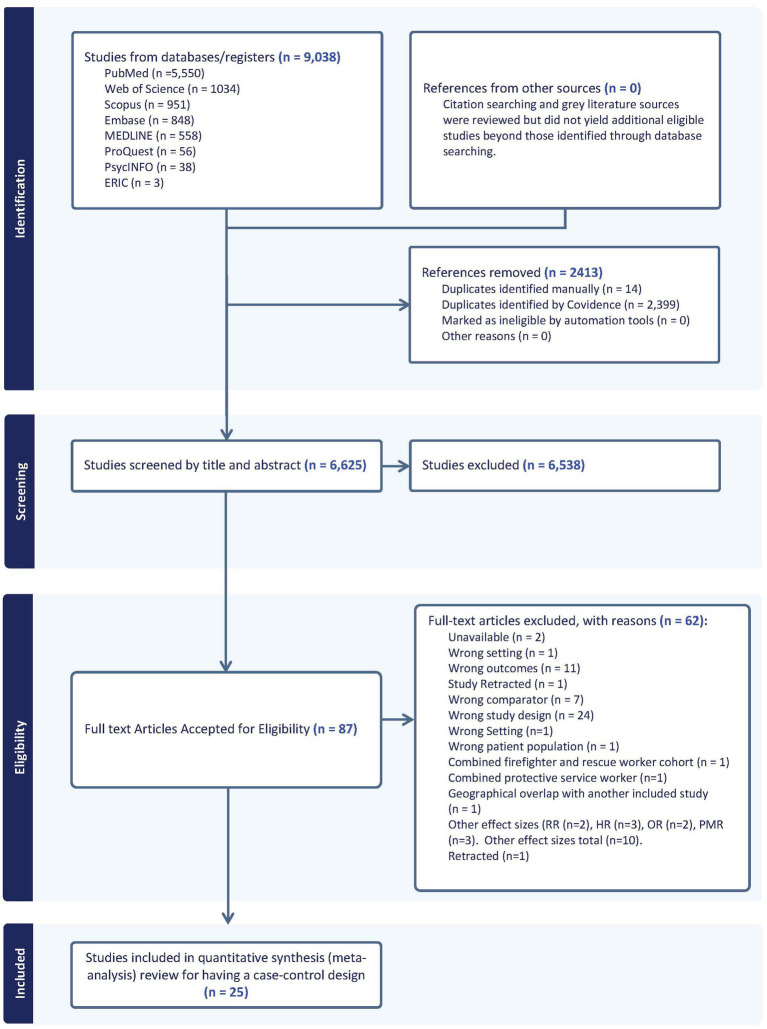
PRISMA flow diagram: non-cancer mortality among firefighters.

Figure 1 displays a PRISMA flow chart outlining the search process and the application of inclusion and exclusion criteria during screening and selection.

### 2.2 Coding of study characteristics

Each study included in the present meta-analysis was revised and coded for: (1) study design characteristics (i.e., cohort, cross-sectional, longitudinal, mixed, and other), (2) cause of death coding system [i.e., International Classification of Diseases (ICD-9 or ICD-10)] (3) source of occupation designations (i.e., employment, certification, cancer, registry, death certificate, and other), (4) type of incidents that firefighters attended, (5) sample characteristics (i.e., age, employment status, gender, race/ethnicity, and smoking status when available), and (6) study characteristics (i.e., location, publication type, and publication year). Two coders independently read the included studies and extracted study information from the 25 studies included in the meta-analysis based on the final coding instrument through REDCap (see the Supplementary material). Any discrepancies discovered in the coding stage were corrected based on the additional review by the last author before data analysis.

### 2.3 Causes of death categories

The following causes of death categories were studied: Heart Disease (ICD-10 codes I00-I09, I11, I13, I20–I51), Accidents (unintentional injuries) (V01–X59, Y85–Y86), Chronic lower respiratory diseases (J40–J47), Cerebrovascular diseases (I60–I69), Diabetes mellitus (E10-E14), Pneumonia/influenza (J09–J18), Intentional self-harm (suicide) (U03, X60–X84, Y87.0), Chronic liver disease and cirrhosis (K70, K73–K74) and equivalent ICD-9 codes.

### 2.4 Effect sizes and standard errors

The primary effect size measure used in the current meta-analysis was the standardized mortality ratio (SMR). The reported SMR value and its associated standard error were directly extracted from each study. When not directly reported, the SMR value was computed by dividing the observed number of firefighters’ mortality due to cause of death of interest by the expected number based on the reference population’s mortality due to the same cause. The computed SMR value was transformed to its logarithm by taking the log of the computed SMR value, whose standard error value was computed by taking a square root of 1/O, where O is the observed number of firefighters’ mortality due to cancer. If only 95% confidence intervals around the reported SMR value are given, the standard error for the log of the SMR value was computed using: (log (SMR_UL) − log(SMR_LL))/(2*1.96), where UL is an upper level of 95% confidence interval, LL is an lower level of 95% confidence interval and SMR is the reported SMR value. The parameter estimates (i.e., pooled SMRE) computed in the meta-analysis were transformed back to the original scale by taking exponential (*e*) to the power of the estimates.

### 2.5 Publication bias

The potential for publication bias, which might occur due to the possibilities that studies demonstrating a significant effect in a favorable direction are more likely to be published, was assessed using multiple indicators. These included: (1) Begg and Mazumdar’s (13) rank correlation test for funnel plot asymmetry, (2) Egger’s et al. (14) regression test of intercept, and (3) funnel plot. When the null hypothesis stating no relationship between effect sizes and their associated precision measures is accepted, we can conclude that there is no sufficient evidence supporting publication bias in the included studies.

### 2.6 Handling dependency in effect sizes

Numerous studies contributed multiple effect sizes from different causes of mortality, which could compromise the assumption of independence required in meta-analysis if examined collectively. To maintain independence among effect sizes, we analyzed the data separately for each mortality source, thereby ensuring that only one independent effect size per mortality cause was included in the analysis.

### 2.7 Study quality assessment

Two content experts independently rated all 25 studies using the Research Triangle Institute (RTI) Item Bank and the Newcastle-Ottawa Scale. Modifications were made to better align with study methods. The Many-facet Rasch Measurement Model (MFRM) was then used to estimate a latent quality score (*z*-score, mean = 0).

### 2.8 Statistical analysis

The metafor package (15) in R version 4.0.2 (R Development Core Team, 2021) was used to analyze the data, which was based on the meta-analytic methods proposed by Hedges and Olkin (16). First, the overall homogeneity was assessed using *Q*-statistics under the assumption that all effects were from the sample population. If the *p*-values of *Q*-statistics were below 0.05, the overall effect was estimated under the random-effects model, where between-study variance was estimated using Restricted Maximum Likelihood (REML) estimation method. Otherwise, the fixed-effects model was used to estimate the overall effect. Second, when between-study variance was found to be significant, a series of moderator analyses with a categorical predictor were performed to explain variation in effect sizes. In particular, the mixed-effects model was adopted for meta-regression when the *p*-value for between-study variation is <0.05 after controlling for the moderator. When the mixed-effects meta-regression model was used, the additional between-study variance after controlling for a moderator that is estimated using the REML method was incorporated. Otherwise, the fixed-effects analysis was used to perform moderator analysis. Moderators used in the current meta-analysis include: (1) participant characteristics (i.e., employment status, gender, race/ethnicity, and smoking status), and (2) study characteristics (i.e., location, publication year, and study quality). More details about random-effects or mixed-effects meta-regression model can be found in Raudenbush (17).

## 3 Results

### 3.1 Description of studies

A total of 25 independent studies published between 1978 and 2025 were selected (Figure 1) and coded in relation to a number of study characteristics including: (1) source of occupation designations (i.e., employment, certification, registry, death certificate, and other), (2) type of incident that firefighters attended (i.e., all, landscape, vehicle, structural, other, mixed, not specified), (3) participant characteristics [e.g., employment status (part-time, full-time, other, not reported, mixed), gender (i.e., male, female, mixed, not reported), race/ethnicity (i.e., white, black, Asian, Hispanic, other, unknown, not reported, mixed), and smoking status (i.e., never smoker, former smoker, current smoker, ever smoker, not reported, mixed)], (4) study characteristics [i.e., location (*k*_study_ = 10 for U.S. studies, *k*_study_ = 15 for non-U.S. studies)] among others (18–42). Ten studies that reported comparisons of non-cancer mortality among firefighters and reference populations using statistical measures other than SMRs were excluded. Table 1 details the characteristic of the included non-cancer causes of death studies. References for the excluded studies are provided in the Supplementary material.

**Table 1:** Characteristics of non-cancer causes of death studies included in meta-analytic analysis.

Record ID	Reference	Firefighter catchment area	Study design	Years of study	Causes of death	# FF and # controls/comparison	Occupational coding source	Data sources	Additional exposure/stratification variables	Statistical measurements	Covariates
1	Pinkerton et al. (38)	United States: San Francisco, Chicago, Philadelphia	Cohort	1950–2009	Leukemia, chronic obstructive pulmonary disease (COPD), cirrhosis & other chronic liver diseases, cerebrovascular diseases, ischemic heart disease	29,992 U.S. career firefighters compared with the U.S. general population	ICD-10	National Death Index-Plus (NDI-Plus), the Social Security Administration Death Master File and the Internal Revenue Service	Employment duration, location of potential exposure, fire-fighting apparatus, number of fire-runs and fire-hours (i.e., the time spent at fires)	Standardized mortality ratios (SMRs)	Gender, age and year at hire, race, birthplace, vital status
2	Petersen et al. (37)	Denmark	Cohort	1970–2014	Endocrine diseases, mental disorders, non-traffic related accidents	11, 775 Danish firefighters compared to the male working population (*n* = 262,168) and the military (*n* = 396,739)	N/A	Supplementary Pension Fund Register, the Danish Civil Registration System, and the Danish Register of Causes of Death	Occupation	Standardized mortality ratios (SMRs)	N/A
3	Guidotti (30)	Alberta, Canada	Cohort	1927–1987	Heart disease, and obstructive pulmonary disease.	3,328 firefighters compared to male residents of the province of Alberta	ICD-9	Alberta Health Care Insurance Plan, death certificates, Canadian Mortality Data Base, Death certificates	Duration of employment, exposure opportunity, or cohort of entry	Standardized mortality ratios (SMRs)	Vital status
4	Baris et al. (21)	Philadelphia, Pennsylvania, United States	Cohort	1926–1986	Nervous system diseases, cerebrovascular diseases, respiratory diseases, genitourinary diseases, all accidents, ischemic heart disease and suicide	7,789 Philadelphia firefighters compared to U.S. white males	ICD-9	Employee Service Record card, the underlying cause of death was abstracted from each death certificate	Years of service, diesel exposure, hire period, age at risk	Standardized mortality ratios (SMRs)	Age (5-year groups) calendar year (5-year groups)
5	Ide (33)	Strathclyde, Scotland	Cohort	1985–1994	Myocardial infarction, road traffic accidents, suicide, musculoskeletal, ocular, injuries, and heart diseases, mental disorders	887 full-time firefighters	N/A	Strathclyde Fire Brigade (SFB)	Years of service	Standardized mortality ratios (SMRs)	N/A
6	Daniels et al. (23)	United States California, Chicago, Philadelphia	Cohort	1950–2009	COPD	29,993 firefighters compared to U.S. population	ICD-10	National Death Index-Plus (NDI-Plus), the Social Security Administration Death Master File (SSA-DMF), personnel and pension board Records, death certificates	Vital status (alive, deceased, unknown causes of death), Employment (avg. hire year, age at hire, employment years, hired before 1950, employed)	Standardized mortality ratios (SMRs)	Race, gender, age
7	Glass et al. (29)	Australia	Cohort	1982–2011	Nervous system, circulatory (hypertensive, ischemic heart disease and cerebrovascular), respiratory (chronic obstructive and pulmonary disease), digestive (diseases of the liver), injury and trauma (all accidents and suicide), other causes	17,394 full-time firefighters compared to 12,663 part-time firefighters	ICD-10	Fire Agencies	Full-time vs. part-time-Incident Type-Duration of employment groups (>3 months <10 years, 10–20 years and 20 + years)-Era first employment (pre-1970, 1970–1994, post-1995)	Standardized mortality ratios (SMRs)	Age (Calendar Year)
8	Glass et al. (27)	Australia	Cohort	1998–2010	Nervous system, circulatory (hypertensive, ischemic heart disease and cerebrovascular), respiratory (chronic obstructive and pulmonary disease), digestive (diseases of the liver), injury and trauma (all accidents and suicide), other causes	Firefighters: 151,546 Firefighters w/ cancer: 11,548 (excluded)	ICD-9 and ICD-10	Various Fire Agencies	Type of volunteer firefighter -Incident types -Number of incidents -Duration of services -Era	Standardized mortality ratios (SMRs)	Age (Calendar Year)
9	Glass et al. (28)	Australia	Cohort	1998–2010	Nervous system, circulatory (ischemic heart disease and cerebrovascular), respiratory (chronic obstructive and pulmonary disease), digestive, injury and trauma (all accidents and suicide), other causes	All female volunteer Firefighters: 16,320 Firefighters cases: 421	ICD-10	Fire Agencies	Employment (paid vs. paid who attended incidents, and volunteer vs. volunteer who attended incidents) -Number of incidents attended (all incidents, all fire incidents, structural fires, landscape fires, vehicle fires) -Era of first service (pre-1970, 1970–1994, post-1995)	Standardized mortality ratios (SMRs)	Age (Calendar Year)
10	Musk et al. (35)	Boston, Massachusetts, United States	Cohort	1915–1975	Infectious disease, diabetes, rheumatic heart disease, chronic nephritis, blood diseases and suicide	2,103 firefighters compared to all Massachusetts males and US white males	ICD-7	Death certificates and Employment records	Active vs. retired	Standardized mortality ratios (SMRs)	N/A
11	Deschamps et al. (25)	Paris, France	Cohort	1977–1991	Cerebrovascular disease	830 firefighters compared to the general French male population	ICD-9	Institut National de la Sante et de la Recherche Medicale (INSERM), Employment records and Pension payment data	Age at death Duration of fire combat Employment	Standardized mortality ratios (SMRs)	N/A
12	Ma et al. (34)	Florida, United States	Cohort	1972–1999	Atherosclerotic heart disease	36,813 firefighters compared to the Florida general population	ICD-9	Office of Vital Statistics of the Florida Department of Health, Certification records	N/A	Standardized mortality ratios (SMRs)	Gender, Age Calendar year
13	Amadeo et al. (18)	France	Cohort	1979–2008	Diseases of skin and subcutaneous tissue, neoplasms, diseases of the genitourinary system, diseases of the digestive system, diseases of the circulatory system, external causes of injury and poisoning, diseases of the nervous system and the sense organs, endocrine, nutritional and metabolic diseases, blood diseases, immunological disorders, Mental and behavioral disorders, diseases of the respiratory system, signs, symptoms and ill-defined causes, infectious and parasitic diseases, congenital malformations and chromosomal abnormalities, diseases of the muscular system/connective tissue	10,829 firefighters compared to the general French male population	N/A	National database containing medical causes of death (INSERM-CépiDC: Institut National de la Santé et de la Recherche Médicale, centre d’épidémiologie sur les causes médicales de décès), Professional staff records	N/A	Standardized mortality ratios (SMRs)	N/A
14	Hansen (31)	Denmark	Cohort	1970–1980	Ischemic heart disease, other diseases, and external causes	886 firefighters compared with other occupational groups from Denmark	ICD-8	Danish National Bureau of Statistics, National Register of Persons, and the National Register of Deaths	N/A	Standardized mortality ratios (SMRs)	Age
15	Rosenstock et al. (39)	United Sates	Cohort	1945–1980	Non-malignant circulatory diseases, non-malignant respiratory diseases, malignant neoplasms of the trachea, bronchus, and lung	4,392 male firefighters compared with the United States population	ICD-8	Department and pension board records, state motor vehicle department records	N/A	Standardized mortality ratios (SMRs)	Age
16	Zhao et al. (42)	Spain	Cohort	2001–2011	Infectious diseases, endocrine diseases, diseases of the nervous system, cardiovascular diseases, diseases of the respiratory system, diseases of the digestive system, urogenital diseases, external causes	27,365 Spanish firefighters compared to the general Spanish population	ICD-10	National follow-up study of Spanish population in the 2001 census	N/A	Standardized mortality ratios (SMRs)	Age
17	Heyer et al. (32)	Seattle, Washington, United States	Cohort	1945–1983	Nervous system disease, circulatory system disease, arteriosclerotic disease, respiratory system disease, acute upper airways, bronchitis, digestive system disease, injury, suicide, homicide	2,289 Seattle Firefighters compared to U.S. white males	N/A	Washington state death records and the National Death Index (NDI)	Time since first employment and duration of employment	Standardized mortality ratios (SMRs)	Age
18	Vena and Fiedler (41)	Buffalo, New York, United States	Cohort	1950–1979	All infective and parasitic disease, benign neoplasms, allergic, endocrine, nutritional diseases, all diseases of nervous system and sense organs, all disease of circulatory system, arteriosclerotic heart disease, all CNS vascular lesions, all respiratory disease, all diseases of the digestive system, cirrhosis of liver, all diseases of genito-urinary system, all external causes, all accidents, suicide	1,867 white male Buffalo firefighters compared to U.S. white males	ICD-8	Death certificates	Calendar year of death, age started working as firefighter, year started working as firefighter, latency (years from onset of work to death), number of years worked as firefighter	Standardized mortality ratios (SMRs)	Age
19	Tornling et al. (40)	Stockholm, Sweden	Cohort	1931–1983	Nervous diseases, circulatory diseases, Ischemic heart disease, cerebrovascular diseases, respiratory diseases, asthma, bronchitis, emphysema. Digestive organs diseases, liver cirrhosis, genitourinary diseases, nephritis, nephros, violent death and poisoning, suicide	1,153 male Stockholm firefighters compared to general Stockholm population	ICD-8	Enrollment records, National Cancer Register, and death certificates	Employment (years), latency (years), fires	Standardized mortality ratios (SMRs)	Age
20	Pedersen et al. (36)	Denmark	Cohort	1970–2021	Infections, endocrine and nutritional disorders, diabetes, mental disorders, nervous system and sensory organs, hypertension, ischemic heart diseases, other heart diseases, cerebrovascular diseases, artery, arteriole and capillary diseases, other circulatory, pneumonia, bronchitis, emphysema and asthma, other respiratory diseases, oral cavity, oesophagus and stomach, liver and bile ducts, other digestive organs, other urinary organs or genitals, skin, bones and connective tissue, senility, symptoms and ill-defined conditions, traffic accidents, non-traffic related accidents, suicide	11,888 male Danish firefighters compared to general working population in Denmark	ICD-10	Danish Civil Registration System and the Danish Register of Causes of Death.	Firefighter employment, specialization, time period of initial employment, employment duration	Standardized mortality ratios (SMRs)	Age, vital status, education marital/cohabiting status and municipality-level residence
21	Eliopulos et al. (26)	Australia	Cohort	1939–1978	Circulatory disease, respiratory disease, accidents, poisonings, and violence	990 male full-time Western Australian firefighters compared to Western Australian men	ICD-8	Western Australia state death registers, Western Australia state electoral roll motor vehicle drivers’ license records, public hospital patient indexes, union records, the telephone directory	Era of fist employment, time since first employment, duration of employment	Standardized mortality ratios (SMRs)	N/A
22	Colbeth et al. (22)	New York, New York, United States	Cohort	2001–2017	Human immunodeficiency virus (HIV), alcoholism, other nervous system diseases, rheumatic heart disease, hypertension with heart disease, ischemic heart disease, cerebrovascular disease, hypertension without heart disease, diseases of the arteries/veins/lymph nodes, pneumonia, chronic obstructive pulmonary disease (COPD), hernia and intestinal obstruction, chronic and unspecified renal failure, motor vehicle, fire in a building, accidental poisoning, intentional self-harm, and assault and homicide	13,266 WTC-exposed Fire Department of the City of New York (FDNY) firefighters compared to the general population	ICD-10	FDNY employee database, self-administered questionnaires completed during routine medical monitoring examinations at FDNY, National Death Index (NDI)	Person-years of follow-up, year hired, WTC exposure level, work assignment, years to death post 9/11/2001	Standardized mortality ratios (SMRs)	Age, race/ethnicity, sex, smoking status, age at death
23	Aronson et al. (20)	Toronto, Canada	Cohort	1950–1989	Infective, parasitic diseases, endocrine, nutritional diseases, circulatory system, respiratory system, digestive system, genitourinary system, symptoms/ill-defined, external causes	5,995 subjects recruited from all six fire departments within Metropolitan Toronto compared to the male Ontario population	ICD-9	Canadian Mortality Data Base (CMDB)	Years of follow-up, years since first employed, years of employment	Standardized mortality ratios (SMRs)	Age
24	Ahn and Jeong (19)	South Korea	Cohort	1980–2007	Infection, endocrine diseases, diabetes, circulatory diseases, ischemic heart diseases, cerebrovascular diseases, respiratory diseases, digestive diseases, liver diseases, injury, poisoning, and external causes, transport accidents, exposure to smoke, fire, and flames, intentional self-harm	29,453 Korean firefighters compared to the Korean male population	ICD-10	National Statistical Office of Korea	Years of initial employment, duration of employment, employment status	Standardized mortality ratios (SMRs)	Age
25	Demers et al. (24)	United States: Seattle, Washington Tacoma, Washington Portland, Oregon	Cohort	1944–1979	Heart disease, ischemic heart disease, other circulatory disease, cerebrovascular disease, diseases of arteries, veins, and pulmonary circulation, respiratory disease, acute upper respiratory diseases, emphysema, asthma, COPS and other respiratory disease	4,546 Seattle, Tacoma, and Portland male firefighters compared to the United States population	ICD-9	National Death Index (NDI)	Currently employed, retired, other alive, deceased, certificates collected, unknown status, years of follow-up	Standardized mortality ratios (SMRs)	Age

### 3.2 Assessing publication bias

**Figure 2:**
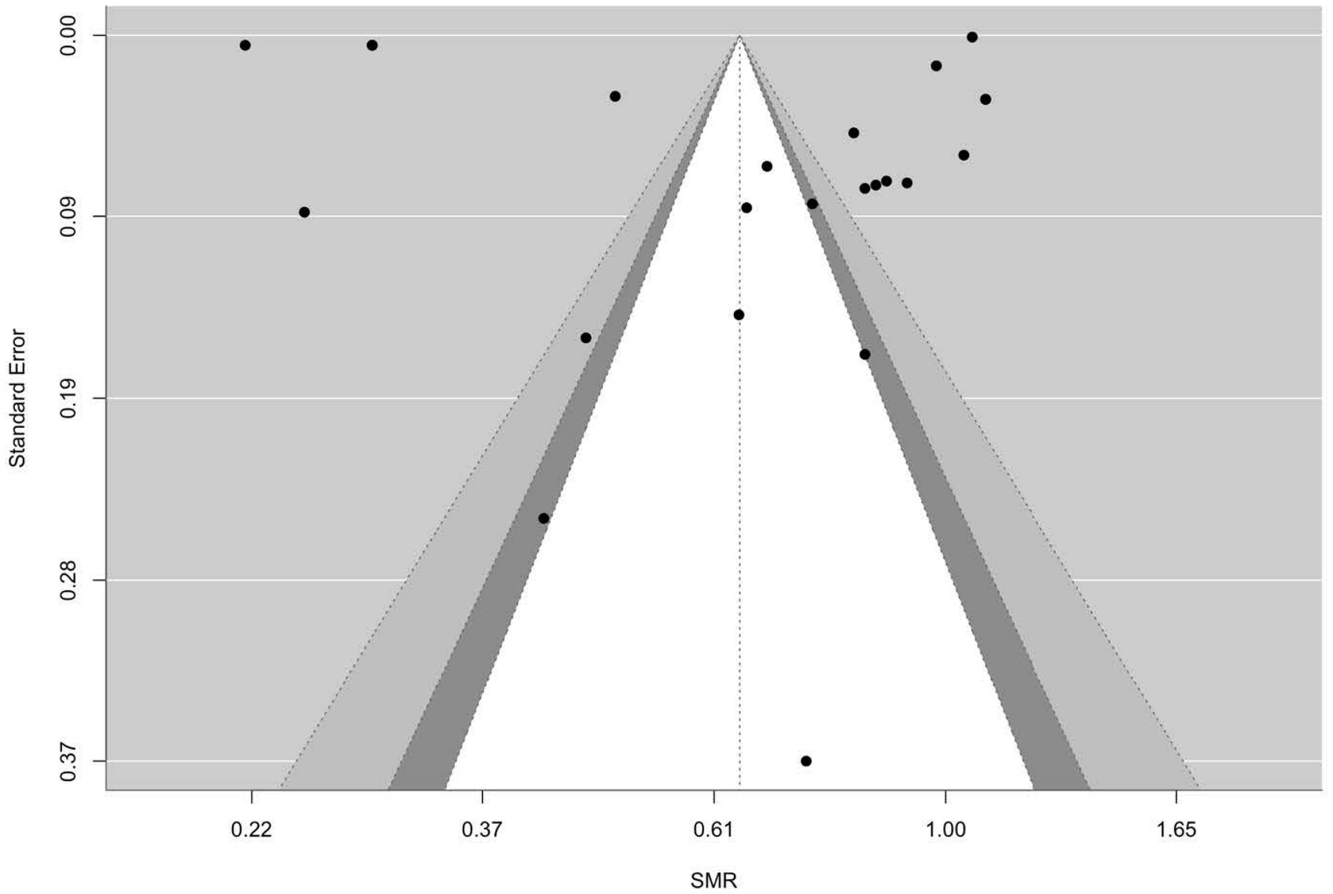
Funnel plots for SMR due to heart disease (I00–I09, I11, I13, I20–I51).

**Figure 3:**
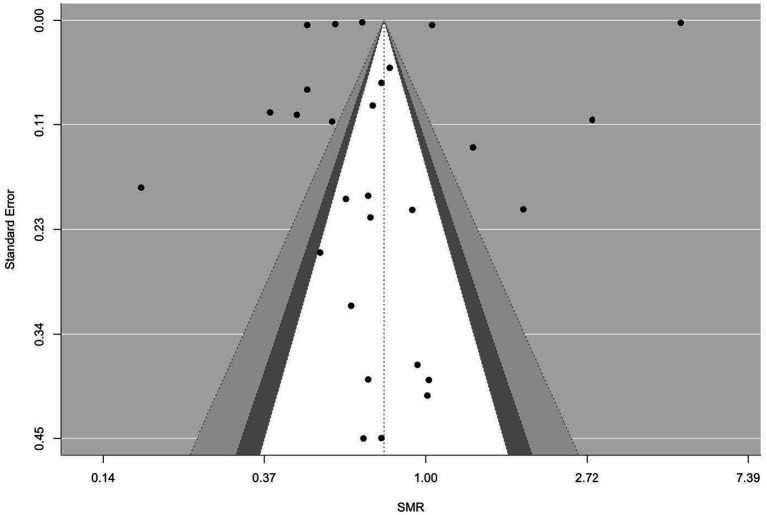
Funnel plots for SMR due to accidents (unintentional injuries) (V01–X59, Y85–Y86).

**Figure 4:**
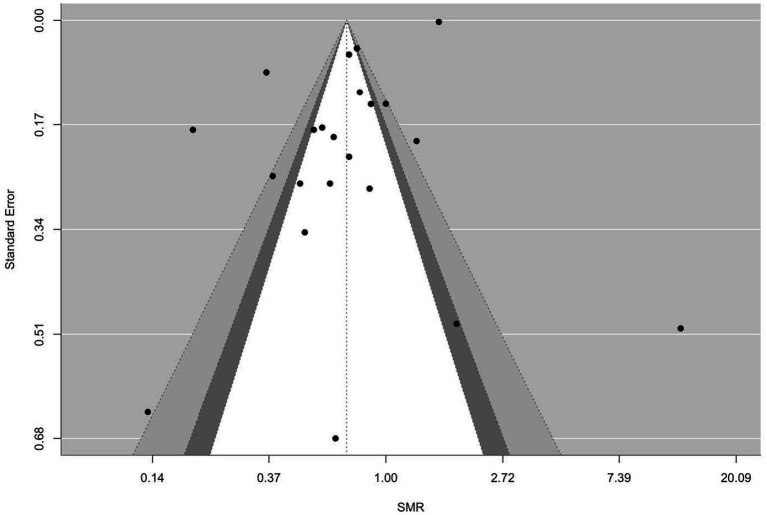
Funnel plots for SMR due to chronic lower respiratory diseases (J40–J47).

**Figure 5:**
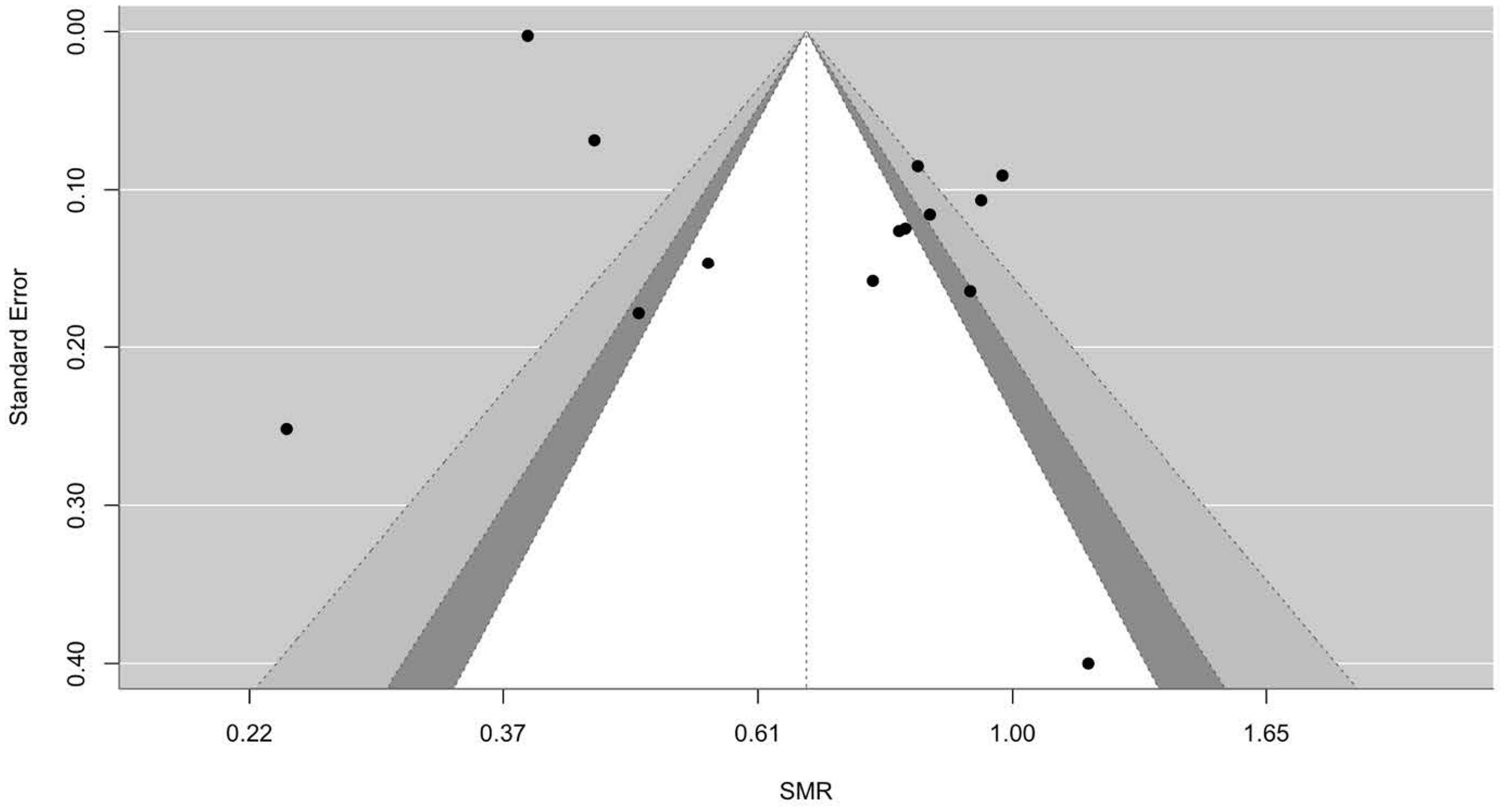
Funnel plots for SMR due to cerebrovascular diseases (I60–169).

**Figure 6:**
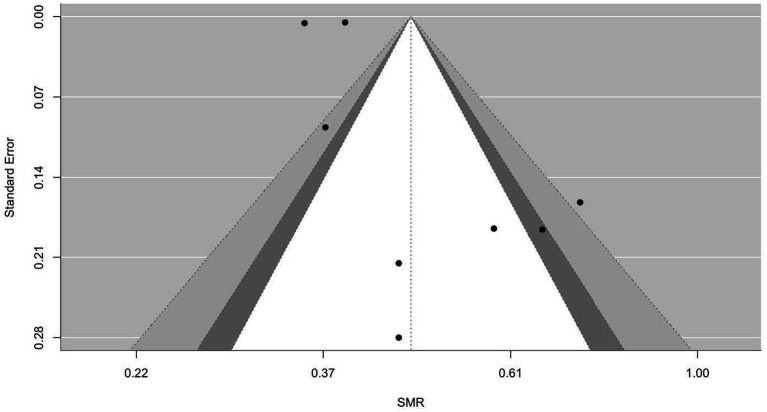
Funnel plots for SMR due to diabetes mellitus (E10–E14).

**Figure 7:**
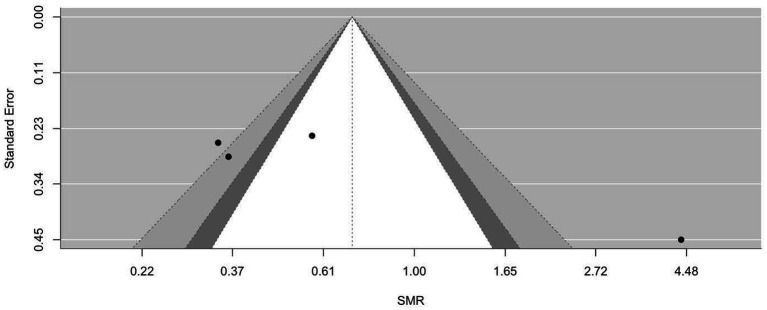
Funnel plots for SMR due to pneumonia/influenza (J09–J18).

**Figure 8:**
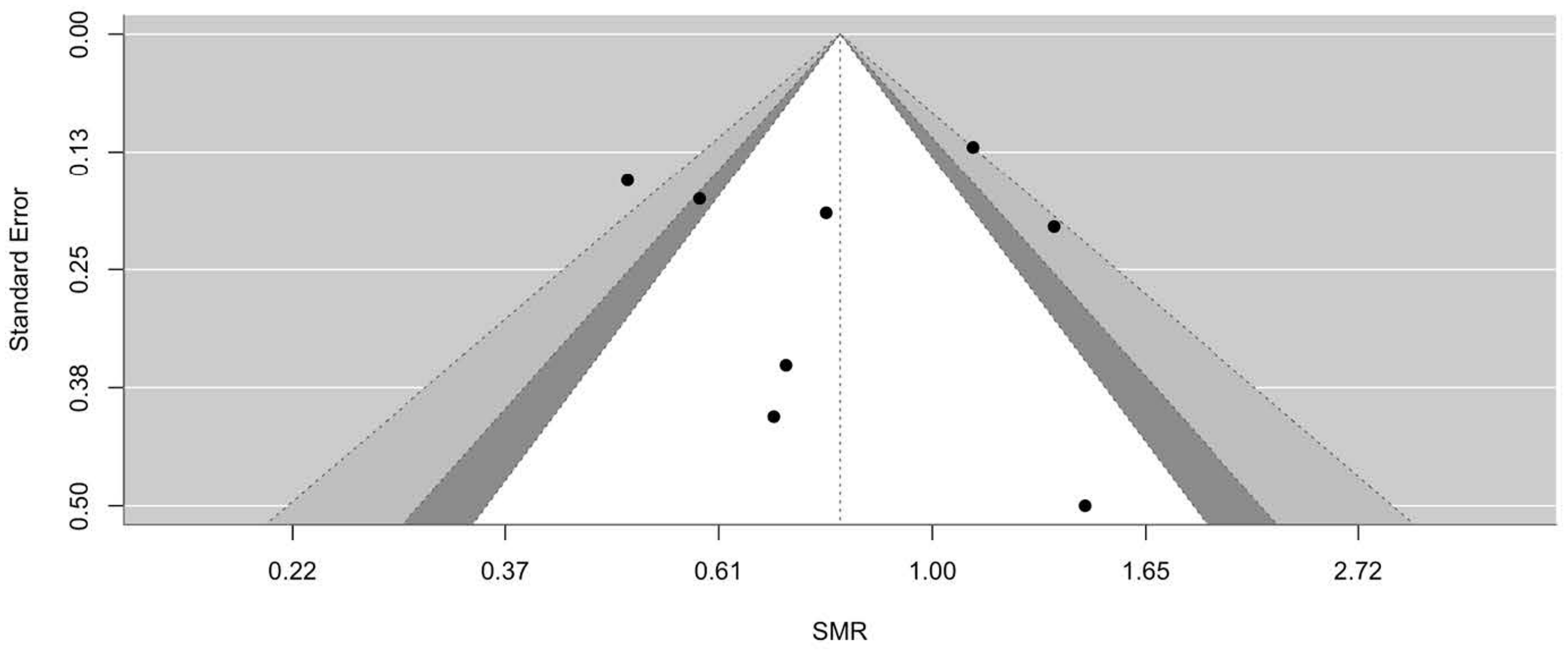
Funnel plots for SMR due to intentional self-harm (suicide) (U03, X60–X84, Y87.0).

**Figure 9:**
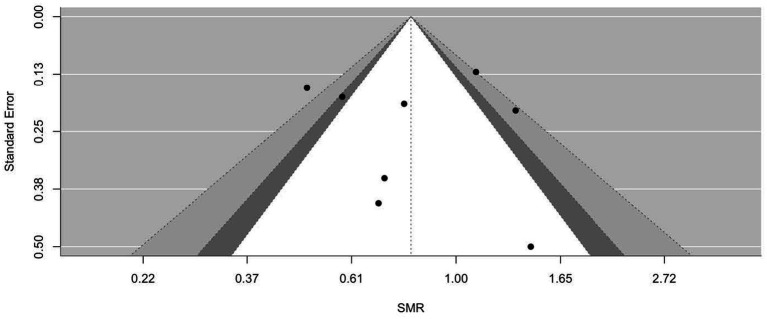
Funnel plots for SMR due to chronic liver disease and cirrhosis (K70, K73–K74).

Figures 2–9 show funnel plots that visually display the relationship between effect sizes and their associated standard errors as measure of precision, separately by the cause of death. Results from (1) Begg and Mazumdar’s rank correlation test for funnel plot asymmetry (*p* = 0.16 for mortality rates due to heart disease; *p* = 0.02 accidents; *p* = 0.40 for chronic lower respiratory disease; *p* = 0.10 for cerebrovascular diseases; *p* = 0.75 for pneumonia/influenza; *p* = 0.002 for intentional self-harm (suicide); *p* = 0.90 for chronic liver disease and cirrhosis; *p* = 0.72 for diabetes mellitus), (2) Egger’s regression test of intercept (*p* = 0.43 for mortality rates due to heart disease; *p* = 0.52 for accidents; *p* = 0.67 for chronic lower respiratory disease; *p* = 0.71 for cerebrovascular diseases; *p* = 0.001 for pneumonia/influenza; *p* = 0.57 for intentional self-harm (suicide); *p* = 0.67 for chronic liver disease and cirrhosis; *p* = 0.01 for diabetes mellitus), and (3) visual inspection of funnel plots indicate that evidence of publication bias varied by cause of death, with no consistent pattern observed across outcomes.

### 3.3 Analysis

#### 3.3.1 Overall analysis

**Figure 10:**
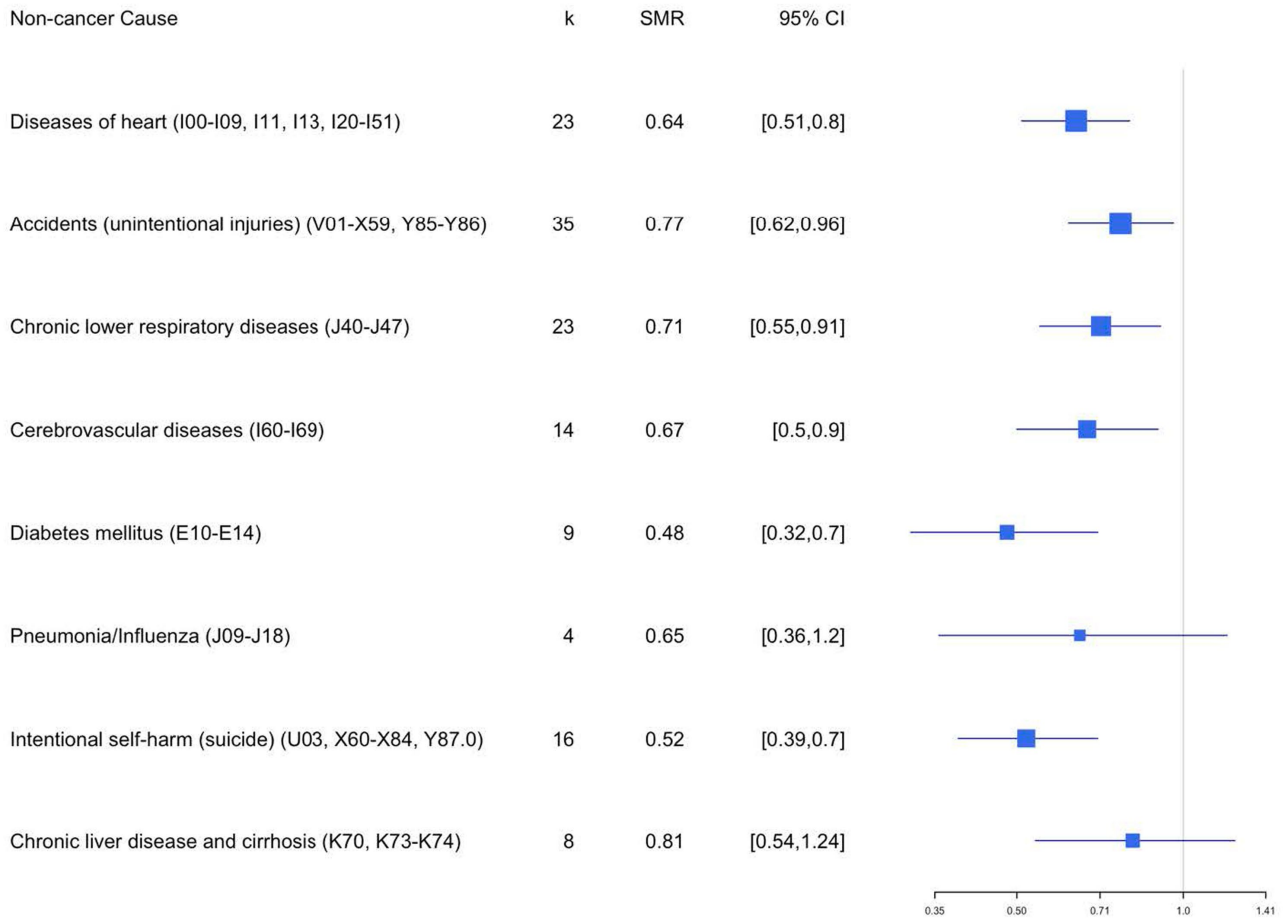
Forest plot for SMR by cause of death.

Table 2 and Figure 10 summarize the estimated overall effects of SMR, separately by mortality source. Results showed firefighters with significantly lower mortality rates due to heart disease (SMRE = 0.64, *k*_smr_ = 23, 95% CI: 0.51–0.81), accidents (SMRE = 0.77, *k*_smr_ = 35, 95% CI: 0.62–0.96), chronic lower respiratory disease (SMRE = 0.71, *k*_smr_ = 23, 95% CI: 0.55–0.91), cerebrovascular diseases (SMRE = 0.67, *k*_smr_ = 14, 95% CI: 0.50–0.90), diabetes mellitus (SMRE = 0.48, *k*_smr_ = 9, 95% CI: 0.32–0.70), intentional self-harm (suicide) (SMRE = 0.52, *k*_smr_ = 16, 95% CI: 0.39–0.70), and chronic liver disease and cirrhosis (SMRE = 0.81, *k*_smr_ = 8, 95% CI: 0.54–1.24) when compared to the reference population. These results suggest that firefighters showed lower mortality due to heart disease by 36%, accidents by 23%, chronic lower respiratory disease by 29% cerebrovascular diseases by 33%, diabetes mellitus by 53%, intentional self-harm (suicide) by 52%, and chronic liver disease and cirrhosis by 19%. However, no significant differences in standardized mortality rates were found by pneumonia/influenza (SMRE = 0.65, *k*_smr_ = 4, 95% CI: 0.36–1.20) as well as between firefighters and reference groups.

**Table 2:** Summary of standardized mortality rate by non-cancer cause among 25 independent studies published during 1978 and 2025.

Cause	SMR	*k*_effect_	*z*	*p*	95% CI	
					LL	UL
Diseases of heart (I00–I09, I11, I13, I20–I51)	0.64^a^	23	−3.88	<0.01	0.51	0.80
Accidents (unintentional injuries) (V01-X59, Y85–Y86)	0.77^b^	35	−2.37	0.02	0.62	0.98
Chronic lower respiratory diseases (J40–J47)	0.71^b^	23	−2.68	0.01	0.55	0.91
Cerebrovascular diseases (I60–I69)	0.67^b^	14	−2.70	0.01	0.50	0.90
Diabetes mellitus (E10–E14)	0.48^a^	9	−3.72	<0.01	0.32	0.70
Pneumonia/Influenza (J09–J18)	0.65	4	−1.38	0.17	0.36	1.20
Intentional self-harm (suicide) (U03, X60–X84, Y87.0)	0.52^a^	16	−4.39	<0.01	0.39	0.70
Chronic liver disease and cirrhosis (K70, K73–K74)	0.81	8	−0.97	0.33	0.54	1.24

a *p* < 0.01.

b *p* < 0.05.

#### 3.3.2 Moderator analysis

Findings from the separate mixed-effects meta-regression models for each cause of death indicate that Standardized Mortality Ratios (SMRs) do not vary significantly according to moderators such as study location, occupation source, type of incident attended by firefighters, gender, race, employment status, smoking status, or quality score. These results suggest that comprehensive meta-regression analyses incorporating all potential moderators were unable to account for the between-study variation in SMRs across datasets segmented by cause of death.

## 4 Discussion

To our knowledge, this is the first meta-analysis to systematically examine standardized mortality ratios (SMRs) for non-cancer causes of death among firefighters, providing a comprehensive assessment of mortality patterns beyond cancer-related outcomes. Overall, firefighter mortality for non-cancer causes generally differs from that of the general population in a cause-specific manner, with predominantly lower mortality but important exceptions. These patterns underscore the need to interpret apparent advantages in the context of occupational selection and the healthy worker effect, while also identifying areas where prevention and surveillance may be strengthened.

This review, spanning 45 years of published literature, shows that firefighters, as an occupational group, exhibit significantly lower mortality rates for multiple non-cancer causes of death compared with the general population. Findings indicate reduced mortality from cardiovascular disease, stroke, unintentional injuries/accidents, respiratory diseases, diabetes, and suicide, while chronic liver disease/cirrhosis and pneumonia/influenza show mortality rates comparable to those of the general population.

The lower mortality from heart disease, stroke, and diabetes likely reflects occupational fitness requirements, ongoing medical surveillance, and the well-known “Healthy Worker Effect” observed in first responders (43). Studies have reported lower prevalence of metabolic syndrome, diabetes, and unfavorable lipid profiles (e.g., high LDL, high triglycerides, low HDL) among firefighters compared to the general population (44–48). Firefighters must meet rigorous physical fitness standards that promote cardiovascular health (49). Regular physical training helps manage blood sugar levels and prevents diabetes-related complications. Additionally, comprehensive medical screenings (50) and wellness programs (51) within fire departments are likely to contribute to early detection and management of cardiovascular and metabolic risk factors. However, a higher prevalence of hypertension among male firefighters (52–54) may increase the risk of sudden cardiac events, particularly during on-duty activities, and warrants further investigation.

Similarly, firefighters show lower mortality from unintentional injuries and accidents compared to the general population, despite these being leading causes of on-duty fatalities. In 2022, over half of firefighter deaths in the U.S. were due to unintentional injuries such as motor vehicle accidents, falls, and structural collapses (6). Rigorous training and safety protocols (55, 56) mitigate these risks, and the lower overall risk suggests that improved safety practices reduce accidental deaths over the life course in comparison to the general population.

Firefighters generally have lower cigarette smoking rates than the general population (2, 7, 57), which positively influence outcomes for heart disease, stroke, and chronic respiratory diseases. Lower mortality from chronic lower respiratory diseases, including COPD, is observed despite routine exposure to smoke, toxic chemicals, and respiratory irritants. Even with protective equipment, long-term exposure to combustion by-products may contribute to respiratory risks. In contrast, although based on fewer studies, mortality from pneumonia/influenza among firefighters is not lower, possibly reflecting occupational exposure to respiratory infections during medical emergency calls and adverse weather conditions (58). Despite vaccine availability, surveys show low influenza vaccination rates among U.S. firefighters (59), underscoring the need for stronger vaccination programs.

The parity in liver disease and cirrhosis mortality may reflect behavioral and occupational factors. Heavy or unhealthy alcohol use, commonly reported among firefighters (60–64) as a coping mechanism for occupational stress, may be a major contributor. However, study numbers were small, confidence intervals wide, and other contributors [e.g., viral hepatitis, expected to be lower due to mandatory hepatitis B vaccination (65), or specific hepatotoxic exposures (66, 67)] remain poorly characterized. Thus, these outcomes remain inconclusive and more studies on liver disease among firefighters are warranted.

The lower suicide rates observed among firefighters may reflect the benefits of camaraderie and social support within the profession. Peer networks serve as strong protective factors against mental health crises (68, 69). Many departments also provide mental health resources and peer support programs to help manage stress and trauma, which have been associated with positive outcomes (70). While prior studies have not found elevated suicide rates, some research suggests higher prevalence of suicidal thoughts (71, 72), emphasizing the need for more representative studies of firefighter mental health.

As noted above, these findings, while at first sight favorable, must be interpreted in light of the healthy worker effect, which is particularly pronounced in physically demanding occupations such as firefighting. Entry and retention require high fitness standards and ongoing health surveillance, while individuals with significant medical conditions are unlikely to enter or remain employed. Comparisons with the general population therefore exaggerate health advantages and underestimate true occupational risks. For example, lower mortality from cardiovascular disease, respiratory disease, diabetes, suicide, and unintentional injuries may partly reflect that the general population includes individuals with chronic illness, disability, or social isolation who would never qualify for or remain in firefighting, thereby inflating apparent health advantages. Our results, therefore, while reflecting differences from the general population baseline, should not be interpreted as direct evidence that firefighting itself is protective.

Ideally, firefighters should be compared with other occupational groups such as police officers, military personnel, or construction workers, which would provide a clearer picture of the specific risks faced by firefighters. However, despite the healthy worker effect, comparison to the general population remains standard in occupational epidemiology and provides a broad baseline for contextualizing mortality patterns.

Our findings support several actionable responses. Patterns of cardiometabolic outcomes support continued annual fitness and medical surveillance into retirement, standardized return-to-duty thresholds for blood pressure and lipid levels, and strengthened pathways for hypertension and statin management. Respiratory infections highlight the need for mandatory annual influenza vaccination with coverage tracking, clear sick-leave policies, and enhanced respiratory protection during periods of increased transmission. Alcohol-related and liver outcomes support department-wide screening for unhealthy alcohol use, brief counseling and referral to treatment, peer-support pathways, and confidential access to care, with consideration of periodic liver enzyme monitoring when appropriate. Injury-related mortality underscores the importance of refreshed driver and operator training, enforcement of seat belt and fall-protection policies, and routine monitoring of near-miss events. Finally, these findings emphasize the need for improved surveillance infrastructure, including retiree registries and comparisons with other occupational groups such as police, military, and construction workers.

This analysis is not without limitations. The limited number of studies for certain non-cancer causes of death (e.g., pneumonia/influenza, liver disease,) warrant more robust analyses for firefighters and these causes of death. Moreover, detailed exposure information was not available (e.g., wildfire versus structural fires, wearing of protective gear, etc.) and therefore was not included. In addition, most research focuses on active-duty personnel, while the prevalence of risk factors, smoking, alcohol use, diet, cholesterol, hypertension, obesity, during retirement remains understudied. This is important because the healthy worker effect is known to decrease with time (73, 74). Thus, surveillance should extend into retirement to capture the full impact of occupational health risks. Furthermore, all studies included here come from North America, Western Europe, and Oceania, limiting generalizability to other regions.

While methodological limitations were proactively addressed such as minimizing Type I error risks through rigorous evaluation of individual study designs for biases and effect size dependencies, and conducting moderator analyses to probe sources of variation including gender, study quality, and effect size metrics, a notable challenge persists in the form of high between-study heterogeneity (*I*^2^ > 90%) across cause-of-death-specific SMR analyses. This heterogeneity endured despite strategic data stratification to address dependencies, use of random-effects models with REML-estimated variances, and mixed-effects meta-regressions incorporating seven predictors (five as dummy variables), which did not yield significant explanations for the remaining variability, likely reflecting unmeasured factors like study population differences, diagnostic criteria, or follow-up durations. By selecting the most independent study effects to avoid regional or temporal overlap, the analysis provides a robust representation of firefighter mortality trends, though multiple publication bias assessments suggest some degree of potential bias that warrants cautious interpretation; future studies can build on this foundation by exploring additional moderators to further illuminate these sources of variation.

## 5 Conclusion

This first meta-analysis of non-cancer causes of death among firefighters provides essential evidence on mortality patterns beyond cancer, while underscoring the complex health profile of this workforce. The substantial reductions in mortality from cardiovascular disease, diabetes, respiratory disease, unintentional injuries, and suicide compared to the general population are real but should not be interpreted as evidence of occupational protection, as they are likely to reflect, at least in part, the healthy worker effect. For liver disease and pneumonia/influenza, the evidence base remains sparse and inconclusive, though not suggestive of elevated risk relative to the general population.

In this context, key priorities are to develop studies using alternative occupational comparators, extend follow-up into retirement, and investigate underexplored exposures such as alcohol use and hepatotoxic agents. While the profession promotes physical fitness and social cohesion, it also presents occupational hazards and lifestyle risks. To better protect firefighter health, future research should focus on exposures in retirement, alcohol use, and toxicological risks. Improved surveillance, targeted interventions, and preventive measures, such as enhanced mental health support, increased vaccination uptake, and stronger safety regulations, remain essential for ensuring the well-being and resilience of firefighters.

## Data Availability

The original contributions presented in the study are included in the article/Supplementary material, further inquiries can be directed to the corresponding author.
