# Supplementary material for: Non-cancer mortality among firefighters: a meta-analytic review of heart disease, stroke, respiratory disease, liver disease, accidents, and suicide
Source: Front Public Health. 2026 Feb 26;14:1714033. doi: 10.3389/fpubh.2026.1714033 (PMC12979482; doi:10.3389/fpubh.2026.1714033)
Supplement: Supplementary file 1 [file Data_Sheet_1.DOCX]

**Non-Cancer Fire MeSH Terms**

(“mortality” OR "cause of death" OR "cause specific mortality" OR “all cause mortality”) AND

(firealarm[All Fields] OR firebomb [All Fields] OR firebombed[All Fields] OR firebombing[All Fields] OR firebombings[All Fields] OR firebreak[All Fields] OR firebreaks[All Fields] OR fireburn[All Fields] OR firebush[All Fields] OR firecenter[All Fields] OR firechief[All Fields] OR firecode[All Fields] OR firedepartment[All Fields] OR “fire department”[All Fields] OR firefighter[All Fields] OR firefighters[All Fields] OR firefighters’[All Fields] OR firefighing[All Fields] OR firefight[All Fields] OR “fire fighter”[All Fields] OR firefighter’[All Fields] OR firefighter’s[All Fields] OR firefighters[All Fields] OR firefighters’[All Fields] OR firefighting[All Fields] OR firefights[All Fields] OR fireground[All Fields] OR firegrounds[All Fields] OR fireguard[All Fields] OR fireguards[All Fields] OR fireguide [All Fields] OR firehouse[All Fields] OR firehouses[All Fields] OR fireman[All Fields] OR fireman**’**[All Fields] OR fireman’s[All Fields] OR firemen[All Fields] OR firemen’s[All Fields] OR fireperson[All Fields] OR firepersons[All Fields] OR firerangers[All Fields] OR fireservice[All Fields] OR “fire service” [All Fields] OR “fire inspector” [All Fields] OR “fire inspectors” [All Fields] OR “fire rescue” [All Fields] OR “firerescue” [All Fields] OR paramedic [All Fields] OR paramedics [All Fields] OR EMT [All Fields] OR “Emergency Medical Technician” [All Fields] OR “Wildland” [All Fields])
