# Supplementary material for: Non-cancer mortality among firefighters: a meta-analytic review of heart disease, stroke, respiratory disease, liver disease, accidents, and suicide
Source: Front Public Health. 2026 Feb 26;14:1714033. doi: 10.3389/fpubh.2026.1714033 (PMC12979482; doi:10.3389/fpubh.2026.1714033)
Supplement: Supplementary file 2 [file Data_Sheet_2.PDF]

Characteristics: Non-cancer Causes of Death

Record ID ______________________

Coder's name
(Please type your last name) ______________________
(please use only lowercase letters)

Study rejected?
◯ Yes
◯ No

Why was it rejected? ______________________

Study Characteristics

Study Id:
Last name of first author (first letter - cap) + first letter (cap) of second author's last name + first letter (cap) of third author's last name (if only two authors exist, just use Last name of first author + first letter of second author's last name)_pubyear
Ahn, Y.S. & Jeong, K.S. 2015 = AhnJ2015
Ahn, Y.S., Jeong, K.S, & Kim, K. S. 2012 = AhnJK2012

if unpublished use 0000 as year ______________________

Publication type
◯ Unpublished
◯ Published
◯ Other

Other (Please list what type...e.g. report, dissertation, government report) ______________________

Please copy and paste the abstract RESULTS section here. ______________________

Study design
◯ Longitudinal
◯ Cross-sectional
◯ Mixed
◯ Case-control
◯ Cohort
◯ Other (Notes)
◯ Not reported

Period of data collection given?
◯ Yes
◯ No

Period of data collection start
Day: ______________________
(please type in DD format)

Period of data collection start<br>Month:	__________________________________<br>(please type in MM format)
Period of data collection start<br>Year:	__________________________________<br>(please type in YYYY format)
Period of data collection end<br>Day:	__________________________________<br>(please type in DD format)
Period of data collection end<br>Month:	__________________________________<br>(please type in MM format)
Period of data collection end<br>Year:	__________________________________<br>(please type in YYYY format)
Information on the controls	____
Control group:<br>Age	☐ At cohort start date<br>☐ At cohort end date<br>☐ At death<br>☐ At diagnosis<br>☐ At hire<br>☐ Not reported
Control group<br>Age at cohort start date: Mean	__________________________________
Control group<br>Age at cohort start date : Standard Deviation	__________________________________<br>(If variance is given please convert to standard deviation)
Control group<br>Age at cohort start date: Min	__________________________________
Control group<br>Age at cohort start date: Max	__________________________________
Control group<br>Age at cohort start<br>Frequencies and percentages	____
Control group<br>Age at cohort end date: Mean	__________________________________
Control group<br>Age at cohort end date : Standard Deviation	__________________________________<br>(If variance is given please convert to standard deviation)

Control group<br>Age at cohort end date: Min	__________________________
Control group<br>Age at cohort end date: Max	__________________________
Control group<br>Age at cohort end<br>Frequencies and percentages	________________________________
Control group<br>Age at death: Mean	__________________________
Control group<br>Age at death : Standard Deviation	__________________________<br>(If variance is given please convert to standard deviation)
Control group<br>Age at death: Min	__________________________
Control group<br>Age at death: Max	__________________________
Control group<br>Age at death<br>Frequencies and percentages	________________________________
Control group<br>Age at dx: Mean	__________________________
Control group<br>Age at dx : Standard Deviation	__________________________<br>(If variance is given please convert to standard deviation)
Control group<br>Age at dx: Min	__________________________
Control group<br>Age at dx: Max	__________________________
Control group<br>Age at dx<br>Frequencies and percentages	________________________________
Control group<br>Age at hire: Mean	__________________________
Control group<br>Age at hire: SD	__________________________<br>(if variance is reported please convert it to standard deviation and type here)
Control group<br>Age at hire: Min	__________________________

Control group
Age at hire: Max
____

Control group
Age at hire
Frequencies and percentages
____

Is duration of employment in years collected categorically or continuously?
- ◯ Categorically
- ◯ Continuous

Control group
Employment in years
Mean:
____

Control group
Employment in years
SD:
____
(if variance is reported please convert it to standard deviation and type here)

Control group
Employment in years
Min:
____

Control group
Employment in years
Max:
____

Type in the categories best fitting for employment in years for the control group
____

Control group
Employment status
- ◯ Part-time
- ◯ Full-time
- ◯ Other (Notes)
- ◯ Not reported

Control group
other status
____

Control Group
Incident type attended
- ☐ All fires
- ☐ Landscape fires
- ☐ Vehicle fires
- ☐ Structural
- ☐ Other (Notes)
- ☐ Not specified

Control group
other incident type
____

Other (Notes)
____

Era of employment:
When they were first certified or first started working as a firefighter so that we can accurately document how long they have been exposed.

Control group<br>Era of first employment	◯ Continuous<br>◯ Categorical
Control group<br>Era of first employment<br>Mean	______________________
Control group<br>Era of employment<br>SD	______________________<br>(if variance is reported please convert it to standard deviation and type here)
Control group<br>Era of employment<br>Minimum=	______________________
Control group<br>Era of employment<br>Maximum	______________________
Type in the categories for Control group<br>Era of employment	____________________________
Control group<br>Gender	☐ Male<br>☐ Female<br>☐ Other (Please specify)<br>☐ Not reported
Other	____________________________
Control group<br>Male n=	______________________
Control group<br>Male %=	______________________
Control group<br>Female n=	______________________
Control group<br>Female %=	______________________
Control group<br>Other n=	______________________
Control group<br>Other %=	______________________

Control group
Race/ Ethnicity

☐ White ☐ Black ☐ Asian ☐ Other ☐ Unknown ☐ Not reported ☐ Notes ☐ Hispanic

Control group
White n= ______________________

Control group
White %= ______________________

Control group
Black n= ______________________

Control group
Black %= ______________________

Control group
Asian n= ______________________

Control group
Asian %= ______________________

Control group
Other ______________________
(please list the other race)

Control group
other n= ______________________

Control group
other %= ______________________

Control group
Unknown n= ______________________

Control group
Unknown %= ______________________

Control group
Hispanic n= ______________________

Control group
Hispanic %= ______________________

Control group race
Notes ______________________

Control group
Smoking Status

○ Never smoker
○ Former smoker
○ Current smoker
○ Ever smoker
○ Not reported

Control group<br>Are the cigarette pack years collected continuously or categorically?	◯ Continuously<br>◯ Categorically<br>◯ Not reported
Control group<br>Cigarette pack years<br>Mean=	__________________________________
Control group<br>Cigarette pack years<br>SD=	__________________________________<br>(if variance is reported please convert it to standard deviation and type here)
Control group<br>Cigarette pack years<br>Min=	__________________________________
Control group<br>Cigarette pack years<br>Max=	__________________________________
Control group<br>Cigarette pack years	◯ < 10 pack years<br>◯ 10-20 pack years<br>◯ 21+ pack years
Location	☐ United States (US)<br>☐ Non-US

Which state(s) are included?

☐ Alabama ☐ Alaska ☐ Arizona ☐ Arkansas ☐ California ☐ Colorado ☐ Connecticut
☐ Delaware ☐ Florida ☐ Georgia ☐ Hawaii ☐ Idaho ☐ Illinois ☐ Indiana ☐ Iowa
☐ Kansas ☐ Kentucky ☐ Louisiana ☐ Maine ☐ Maryland ☐ Massachusetts ☐ Michigan
☐ Minnesota ☐ Mississippi ☐ Missouri ☐ Montana ☐ Nebraska ☐ Nevada ☐ New Hampshire
☐ New Jersey ☐ New Mexico ☐ New York ☐ North Carolina ☐ North Dakota ☐ Ohio
☐ Oklahoma ☐ Oregon ☐ Pennsylvania ☐ Rhode Island ☐ South Carolina ☐ South Dakota
☐ Tennessee ☐ Texas ☐ Utah ☐ Vermont ☐ Virginia ☐ Washington ☐ West Virginia
☐ Wisconsin ☐ Wyoming

Which cities in Alabama?

☐ Alabaster ☐ Albertville ☐ Alexander City ☐ Andalusia ☐ Anniston ☐ Arab ☐ Athens
☐ Atmore ☐ Attalla ☐ Auburn ☐ Bay Minette ☐ Bessemer ☐ Birmingham ☐ Boaz
☐ Center Point ☐ Chickasaw ☐ Clanton ☐ Cullman ☐ Daphne ☐ Decatur ☐ Demopolis
☐ Dothan ☐ Enterprise ☐ Eufaula ☐ Fairfield ☐ Fairhope ☐ Florence ☐ Foley
☐ Forestdale ☐ Fort Payne ☐ Fort Rucker ☐ Fultondale ☐ Gadsden ☐ Gardendale
☐ Greenville ☐ Guntersville ☐ Hamilton ☐ Hartselle ☐ Helena ☐ Homewood
☐ Hoover ☐ Hueytown ☐ Huntsville ☐ Irondale ☐ Jacksonville ☐ Jasper ☐ Lanett
☐ Leeds ☐ Madison ☐ Millbrook ☐ Mobile ☐ Monroeville ☐ Montgomery ☐ Moody
☐ Mountain Brook ☐ Muscle Shoals ☐ Northport ☐ Opelika ☐ Opp ☐ Oxford
☐ Ozark ☐ Pelham ☐ Pell City ☐ Phenix City ☐ Pleasant Grove ☐ Prattville ☐ Prichard
☐ Rainbow City ☐ Roanoke ☐ Russellville ☐ Saks ☐ Saraland ☐ Scottsboro ☐ Selma
☐ Sheffield ☐ Smiths ☐ Southside ☐ Sylacauga ☐ Talladega ☐ Tarrant ☐ Theodore
☐ Tillmans Corner ☐ Troy ☐ Trussville ☐ Tuscaloosa ☐ Tuscumbia ☐ Tuskegee
☐ Valley ☐ Vestavia Hills ☐ Not listed

Which cities in Alaska?

☐ Anchorage ☐ Cordova ☐ Fairbanks ☐ Haines ☐ Homer ☐ Juneau ☐ Ketchikan
☐ Kodiak ☐ Kotzebue ☐ Nome ☐ Palmer ☐ Seward ☐ Sitka ☐ Skagway ☐ Valdez
☐ Not listed

Which cities in Arizona?

☐ Alexander City ☐ Andalusia ☐ Anniston ☐ Athens ☐ Atmore ☐ Auburn ☐ Bessemer
☐ Birmingham ☐ Chickasaw ☐ Clanton ☐ Cullman ☐ Decatur ☐ Demopolis ☐ Dothan
☐ Enterprise ☐ Eufaula ☐ Florence ☐ Fort Payne ☐ Gadsden ☐ Greenville ☐ Guntersville
☐ Huntsville ☐ Jasper ☐ Marion ☐ Mobile ☐ Montgomery ☐ Opelika ☐ Ozark
☐ Phenix City ☐ Prichard ☐ Scottsboro ☐ Selma ☐ Sheffield ☐ Sylacauga ☐ Talladega
☐ Troy ☐ Tuscaloosa ☐ Tuscumbia ☐ Tuskegee ☐ Not listed

Which cities in Arkansas?

☐ Arkadelphia ☐ Arkansas Post ☐ Batesville ☐ Benton ☐ Blytheville ☐ Camden
☐ Conway ☐ Crossett ☐ El Dorado ☐ Fayetteville ☐ Forrest City ☐ Fort Smith
☐ Harrison ☐ Helena ☐ Hope ☐ Hot Springs ☐ Jacksonville ☐ Jonesboro ☐ Little Rock
☐ Magnolia ☐ Morrilton ☐ Newport ☐ North Little Rock ☐ Osceola ☐ Pine Bluff
☐ Rogers ☐ Searcy ☐ Stuttgart ☐ Van Buren ☐ West Memphis ☐ Not listed

Which cities in California?

☐ Alameda ☐ Alhambra ☐ Anaheim ☐ Antioch ☐ Arcadia ☐ Bakersfield ☐ Barstow
☐ Belmont ☐ Berkeley ☐ Beverly Hills ☐ Brea ☐ Buena Park ☐ Burbank ☐ Calexico
☐ Calistoga ☐ Carlsbad ☐ Carmel ☐ Chico ☐ Chula Vista ☐ Claremont ☐ Compton
☐ Concord ☐ Corona ☐ Coronado ☐ Costa Mesa ☐ Culver City ☐ Daly City ☐ Davis
☐ Downey ☐ El Centro ☐ El Cerrito ☐ El Monte ☐ Escondido ☐ Eureka ☐ Fairfield
☐ Fontana ☐ Fremont ☐ Fresno ☐ Fullerton ☐ Garden Grove ☐ Glendale ☐ Hayward
☐ Hollywood ☐ Huntington Beach ☐ Indio ☐ Inglewood ☐ Irvine ☐ La Habra
☐ Laguna Beach ☐ Lancaster ☐ Livermore ☐ Lodi ☐ Lompoc ☐ Long Beach ☐ Los Angeles
☐ Malibu ☐ Martinez ☐ Marysville ☐ Menlo Park ☐ Merced ☐ Modesto ☐ Monterey
☐ Mountain View ☐ Napa ☐ Needles ☐ Newport Beach ☐ Norwalk ☐ Novato
☐ Oakland ☐ Oceanside ☐ Ojai ☐ Ontario ☐ Orange ☐ Oroville ☐ Oxnard
☐ Pacific Grove ☐ Palm Springs ☐ Palmdale ☐ Palo Alto ☐ Pasadena ☐ Petaluma
☐ Pomona ☐ Port Hueneme ☐ Rancho Cucamonga ☐ Red Bluff ☐ Redding ☐ Redlands
☐ Redondo Beach ☐ Redwood City ☐ Richmond ☐ Riverside ☐ Roseville ☐ Sacramento
☐ Salinas ☐ San Bernardino ☐ San Clemente ☐ San Diego ☐ San Fernando ☐ San Francisco
☐ San Gabriel ☐ San Jose ☐ San Juan Capistrano ☐ San Leandro ☐ San Luis Obispo
☐ San Marino ☐ San Mateo ☐ San Pedro ☐ San Rafael ☐ San Simeon ☐ Santa Ana
☐ Santa Barbara ☐ Santa Clara ☐ Santa Clarita ☐ Santa Cruz ☐ Santa Monica ☐ Santa Rosa
☐ Sausalito ☐ Simi Valley ☐ Sonoma ☐ South San Francisco ☐ Stockton ☐ Sunnyvale
☐ Susanville ☐ Thousand Oaks ☐ Torrance ☐ Turlock ☐ Ukiah ☐ Vallejo ☐ Ventura
☐ Victorville ☐ Visalia ☐ Walnut Creek ☐ Watts ☐ West Covina ☐ Whittier ☐ Woodland
☐ Yorba Linda ☐ Yuba City ☐ Not listed

Which cities in Colorado?

☐ Alamosa ☐ Aspen ☐ Aurora ☐ Boulder ☐ Breckenridge ☐ Brighton ☐ Canon City
☐ Central City ☐ Climax ☐ Colorado Springs ☐ Cortez ☐ Cripple Creek ☐ Denver
☐ Durango ☐ Englewood ☐ Estes Park ☐ Fort Collins ☐ Fort Morgan ☐ Georgetown
☐ Glenwood Springs ☐ Golden ☐ Grand Junction ☐ Greeley ☐ Gunnison ☐ La Junta
☐ Leadville ☐ Littleton ☐ Longmont ☐ Loveland ☐ Montrose ☐ Ouray ☐ Pagosa Springs
☐ Pueblo ☐ Silverton ☐ Steamboat Springs ☐ Sterling ☐ Telluride ☐ Trinidad
☐ Vail ☐ Walsenburg ☐ Westminster ☐ Not listed

Which cities in Connecticut?

☐ Ansonia ☐ Berlin ☐ Bloomfield ☐ Branford ☐ Bridgeport ☐ Bristol ☐ Coventry
☐ Danbury ☐ Darien ☐ Derby ☐ East Hartford ☐ East Haven ☐ Enfield ☐ Fairfield
☐ Farmington ☐ Greenwich ☐ Groton ☐ Guilford ☐ Hamden ☐ Hartford ☐ Lebanon
☐ Litchfield ☐ Manchester ☐ Mansfield ☐ Meriden ☐ Middletown ☐ Milford ☐ Mystic
☐ Naugatuck ☐ New Britain ☐ New Haven ☐ New London ☐ North Haven ☐ Norwalk
☐ Norwich ☐ Old Saybrook ☐ Orange ☐ Seymour ☐ Shelton ☐ Simsbury ☐ Southington
☐ Stamford ☐ Stonington ☐ Stratford ☐ Torrington ☐ Wallingford ☐ Waterbury
☐ Waterford ☐ Watertown ☐ West Hartford ☐ West Haven ☐ Westport ☐ Wethersfield
☐ Willimantic ☐ Windham ☐ Windsor ☐ Windsor Locks ☐ Winsted ☐ Not listed

Which cities in Delaware?

☐ Dover ☐ Lewes ☐ Milford ☐ New Castle ☐ Newark ☐ Smyrna ☐ Wilmington
☐ Not listed

Which cities in Florida?

☐ Apalachicola ☐ Bartow ☐ Belle Glade ☐ Boca Raton ☐ Bradenton ☐ Cape Coral
☐ Clearwater ☐ Cocoa Beach ☐ Cocoa-Rockledge ☐ Coral Gables ☐ Daytona Beach
☐ De Land ☐ Deerfield Beach ☐ Delray Beach ☐ Fernandina Beach ☐ Fort Lauderdale
☐ Fort Myers ☐ Fort Pierce ☐ Fort Walton Beach ☐ Gainesville ☐ Hallandale Beach
☐ Hialeah ☐ Hollywood ☐ Homestead ☐ Jacksonville ☐ Key West ☐ Lake City
☐ Lake Wales ☐ Lakeland ☐ Largo ☐ Melbourne ☐ Miami ☐ Miami Beach ☐ Naples
☐ New Smyrna Beach ☐ Ocala ☐ Orlando ☐ Ormond Beach ☐ Palatka ☐ Palm Bay
☐ Palm Beach ☐ Panama City ☐ Pensacola ☐ Pompano Beach ☐ Saint Augustine
☐ Saint Petersburg ☐ Sanford ☐ Sarasota ☐ Sebring ☐ Tallahassee ☐ Tampa
☐ Tarpon Springs ☐ Titusville ☐ Venice ☐ West Palm Beach ☐ White Springs ☐ Winter Haven
☐ Winter Park ☐ Not listed

Which cities in Georgia?

☐ Albany ☐ Americus ☐ Andersonville ☐ Athens ☐ Atlanta ☐ Augusta ☐ Bainbridge
☐ Blairsville ☐ Brunswick ☐ Calhoun ☐ Carrollton ☐ Columbus ☐ Dahlonega
☐ Dalton ☐ Darien ☐ Decatur ☐ Douglas ☐ East Point ☐ Fitzgerald ☐ Fort Valley
☐ Gainesville ☐ La Grange ☐ Macon ☐ Marietta ☐ Milledgeville ☐ Plains ☐ Rome
☐ Savannah ☐ Toccoa ☐ Valdosta ☐ Warm Springs ☐ Warner Robins ☐ Washington
☐ Waycross ☐ Not listed

Which cities in Hawaii?

☐ Hanalei ☐ Hilo ☐ Honaunau ☐ Honolulu ☐ Kahului ☐ Kaneohe ☐ Kapaa
☐ Kawaihae ☐ Lahaina ☐ Laie ☐ Wahiawa ☐ Wailuku ☐ Waimea ☐ Not listed

Which cities in Idaho?

☐ Blackfoot ☐ Boise ☐ Bonners Ferry ☐ Caldwell ☐ Coeur d'Alene ☐ Idaho City
☐ Idaho Falls ☐ Kellogg ☐ Lewiston ☐ Moscow ☐ Nampa ☐ Pocatello ☐ Priest River
☐ Rexburg ☐ Sun Valley ☐ Twin Falls ☐ Not listed

Which cities in Illinois?

☐ Alton ☐ Arlington Heights ☐ Arthur ☐ Aurora ☐ Belleville ☐ Belvidere ☐ Bloomington
☐ Brookfield ☐ Cahokia ☐ Cairo ☐ Calumet City ☐ Canton ☐ Carbondale ☐ Carlinville
☐ Carthage ☐ Centralia ☐ Champaign ☐ Charleston ☐ Chester ☐ Chicago ☐ Chicago Heights
☐ Cicero ☐ Collinsville ☐ Danville ☐ Decatur ☐ DeKalb ☐ Des Plaines ☐ Dixon
☐ East Moline ☐ East Saint Louis ☐ Effingham ☐ Elgin ☐ Elmhurst ☐ Evanston
☐ Freeport ☐ Galena ☐ Galesburg ☐ Glen Ellyn ☐ Glenview ☐ Granite City ☐ Harrisburg
☐ Herrin ☐ Highland Park ☐ Jacksonville ☐ Joliet ☐ Kankakee ☐ Kaskaskia ☐ Kewanee
☐ La Salle ☐ Lake Forest ☐ Libertyville ☐ Lincoln ☐ Lisle ☐ Lombard ☐ Macomb
☐ Mattoon ☐ Moline ☐ Monmouth ☐ Mount Vernon ☐ Mundelein ☐ Naperville
☐ Nauvoo ☐ Normal ☐ North Chicago ☐ Oak Park ☐ Oregon ☐ Ottawa ☐ Palatine
☐ Park Forest ☐ Park Ridge ☐ Pekin ☐ Peoria ☐ Petersburg ☐ Pontiac ☐ Quincy
☐ Rantoul ☐ River Forest ☐ Rock Island ☐ Rockford ☐ Salem ☐ Shawneetown
☐ Skokie ☐ South Holland ☐ Springfield ☐ Streator ☐ Summit ☐ Urbana ☐ Vandalia
☐ Virden ☐ Waukegan ☐ Wheaton ☐ Wilmette ☐ Winnetka ☐ Wood River ☐ Zion
☐ Not listed

Which cities in Indiana

☐ Anderson ☐ Bedford ☐ Bloomington ☐ Columbus ☐ Connersville ☐ Corydon
☐ Crawfordsville ☐ East Chicago ☐ Elkhart ☐ Elwood ☐ Evansville ☐ Fort Wayne
☐ French Lick ☐ Gary ☐ Geneva ☐ Goshen ☐ Greenfield ☐ Hammond ☐ Hobart
☐ Huntington ☐ Indianapolis ☐ Jeffersonville ☐ Kokomo ☐ Lafayette ☐ Madison
☐ Marion ☐ Michigan City ☐ Mishawaka ☐ Muncie ☐ Nappanee ☐ Nashville ☐ New Albany
☐ New Castle ☐ New Harmony ☐ Peru ☐ Plymouth ☐ Richmond ☐ Santa Claus
☐ Shelbyville ☐ South Bend ☐ Terre Haute ☐ Valparaiso ☐ Vincennes ☐ Wabash
☐ West Lafayette ☐ Not listed

Which cities in Iowa?

☐ Amana Colonies ☐ Ames ☐ Boone ☐ Burlington ☐ Cedar Falls ☐ Cedar Rapids
☐ Charles City ☐ Cherokee ☐ Clinton ☐ Council Bluffs ☐ Davenport ☐ Des Moines
☐ Dubuque ☐ Estherville ☐ Fairfield ☐ Fort Dodge ☐ Grinnell ☐ Indianola ☐ Iowa City
☐ Keokuk ☐ Mason City ☐ Mount Pleasant ☐ Muscatine ☐ Newton ☐ Oskaloosa
☐ Ottumwa ☐ Sioux City ☐ Waterloo ☐ Webster City ☐ West Des Moines ☐ Not listed

Which cities in Kansas?

☐ Abilene ☐ Arkansas City ☐ Atchison ☐ Chanute ☐ Coffeyville ☐ Council Grove
☐ Dodge City ☐ Emporia ☐ Fort Scott ☐ Garden City ☐ Great Bend ☐ Hays ☐ Hutchinson
☐ Independence ☐ Junction City ☐ Kansas City ☐ Lawrence ☐ Leavenworth ☐ Liberal
☐ Manhattan ☐ McPherson ☐ Medicine Lodge ☐ Newton ☐ Olathe ☐ Osawatomie
☐ Ottawa ☐ Overland Park ☐ Pittsburg ☐ Salina ☐ Shawnee ☐ Smith Center
☐ Topeka ☐ Wichita ☐ Not listed

Which cities in Kentucky?

☐ Ashland ☐ Barbourville ☐ Bardstown ☐ Berea ☐ Boonesborough ☐ Bowling Green
☐ Campbellsville ☐ Covington ☐ Danville ☐ Elizabethtown ☐ Frankfort ☐ Harlan
☐ Harrodsburg ☐ Hazard ☐ Henderson ☐ Hodgenville ☐ Hopkinsville ☐ Lexington
☐ Louisville ☐ Mayfield ☐ Maysville ☐ Middlesboro ☐ Newport ☐ Owensboro
☐ Paducah ☐ Paris ☐ Richmond ☐ Not listed

Which cities in Louisiana?

☐ Abbeville ☐ Alexandria ☐ Bastrop ☐ Baton Rouge ☐ Bogalusa ☐ Bossier City
☐ Gretna ☐ Houma ☐ Lafayette ☐ Lake Charles ☐ Monroe ☐ Morgan City ☐ Natchitoches
☐ New Iberia ☐ New Orleans ☐ Opelousas ☐ Ruston ☐ Saint Martinville ☐ Shreveport
☐ Thibodaux ☐ Not listed

Which cities in Maine?

☐ Auburn ☐ Augusta ☐ Bangor ☐ Bar Harbor ☐ Bath ☐ Belfast ☐ Biddeford
☐ Boothbay Harbor ☐ Brunswick ☐ Calais ☐ Caribou ☐ Castine ☐ Eastport ☐ Ellsworth
☐ Farmington ☐ Fort Kent ☐ Gardiner ☐ Houlton ☐ Kennebunkport ☐ Kittery
☐ Lewiston ☐ Lubec ☐ Machias ☐ Orono ☐ Portland ☐ Presque Isle ☐ Rockland
☐ Rumford ☐ Saco ☐ Scarborough ☐ Waterville ☐ York ☐ Not listed

Which cities in Maryland?

☐ Aberdeen ☐ Annapolis ☐ Baltimore ☐ Bethesda-Chevy Chase ☐ Bowie ☐ Cambridge
☐ Catonsville ☐ College Park ☐ Columbia ☐ Cumberland ☐ Easton ☐ Elkton
☐ Emmitsburg ☐ Frederick ☐ Greenbelt ☐ Hagerstown ☐ Hyattsville ☐ Laurel
☐ Oakland ☐ Ocean City ☐ Rockville ☐ Saint Marys City ☐ Salisbury ☐ Silver Spring
☐ Takoma Park ☐ Towson ☐ Westminster ☐ Not listed

Which cities in Massachusetts?

☐ Abington ☐ Adams ☐ Amesbury ☐ Amherst ☐ Andover ☐ Arlington ☐ Athol
☐ Attleboro ☐ Barnstable ☐ Bedford ☐ Beverly ☐ Boston ☐ Bourne ☐ Braintree
☐ Brockton ☐ Brookline ☐ Cambridge ☐ Canton ☐ Charlestown ☐ Chelmsford
☐ Chelsea ☐ Chicopee ☐ Clinton ☐ Cohasset ☐ Concord ☐ Danvers ☐ Dartmouth
☐ Dedham ☐ Dennis ☐ Duxbury ☐ Eastham ☐ Edgartown ☐ Everett ☐ Fairhaven
☐ Fall River ☐ Falmouth ☐ Fitchburg ☐ Framingham ☐ Gloucester ☐ Great Barrington
☐ Greenfield ☐ Groton ☐ Harwich ☐ Haverhill ☐ Hingham ☐ Holyoke ☐ Hyannis
☐ Ipswich ☐ Lawrence ☐ Lenox ☐ Leominster ☐ Lexington ☐ Lowell ☐ Ludlow
☐ Lynn ☐ Malden ☐ Marblehead ☐ Marlborough ☐ Medford ☐ Milton ☐ Nahant
☐ Natick ☐ New Bedford ☐ Newburyport ☐ Newton ☐ North Adams ☐ Northampton
☐ Norton ☐ Norwood ☐ Peabody ☐ Pittsfield ☐ Plymouth ☐ Provincetown ☐ Quincy
☐ Randolph ☐ Revere ☐ Salem ☐ Sandwich ☐ Saugus ☐ Somerville ☐ South Hadley
☐ Springfield ☐ Stockbridge ☐ Stoughton ☐ Sturbridge ☐ Sudbury ☐ Taunton
☐ Tewksbury ☐ Truro ☐ Watertown ☐ Webster ☐ Wellesley ☐ Wellfleet ☐ West Bridgewater
☐ West Springfield ☐ Westfield ☐ Weymouth ☐ Whitman ☐ Williamstown ☐ Woburn
☐ Woods Hole ☐ Worcester ☐ Not listed

Which cities in Michigan?

☐ Adrian ☐ Alma ☐ Ann Arbor ☐ Battle Creek ☐ Bay City ☐ Benton Harbor ☐ Bloomfield Hills
☐ Cadillac ☐ Charlevoix ☐ Cheboygan ☐ Dearborn ☐ Detroit ☐ East Lansing
☐ Eastpointe ☐ Ecorse ☐ Escanaba ☐ Flint ☐ Grand Haven ☐ Grand Rapids ☐ Grayling
☐ Grosse Pointe ☐ Hancock ☐ Highland Park ☐ Holland ☐ Houghton ☐ Interlochen
☐ Iron Mountain ☐ Ironwood ☐ Ishpeming ☐ Jackson ☐ Kalamazoo ☐ Lansing
☐ Livonia ☐ Ludington ☐ Mackinaw City ☐ Manistee ☐ Marquette ☐ Menominee
☐ Midland ☐ Monroe ☐ Mount Clemens ☐ Mount Pleasant ☐ Muskegon ☐ Niles
☐ Petoskey ☐ Pontiac ☐ Port Huron ☐ Royal Oak ☐ Saginaw ☐ Saint Ignace
☐ Saint Joseph ☐ Sault Sainte Marie ☐ Traverse City ☐ Trenton ☐ Warren ☐ Wyandotte
☐ Ypsilanti ☐ Not listed

Which cities in Minnesota?

☐ Albert Lea ☐ Alexandria ☐ Austin ☐ Bemidji ☐ Bloomington ☐ Brainerd ☐ Crookston
☐ Duluth ☐ Ely ☐ Eveleth ☐ Faribault ☐ Fergus Falls ☐ Hastings ☐ Hibbing
☐ International Falls ☐ Little Falls ☐ Mankato ☐ Minneapolis ☐ Moorhead ☐ New Ulm
☐ Northfield ☐ Owatonna ☐ Pipestone ☐ Red Wing ☐ Rochester ☐ Saint Cloud
☐ Saint Paul ☐ Sauk Centre ☐ South Saint Paul ☐ Stillwater ☐ Virginia ☐ Willmar
☐ Winona

Which cities in Mississippi?

☐ Bay Saint Louis ☐ Biloxi ☐ Canton ☐ Clarksdale ☐ Columbia ☐ Columbus ☐ Corinth
☐ Greenville ☐ Greenwood ☐ Grenada ☐ Gulfport ☐ Hattiesburg ☐ Holly Springs
☐ Jackson ☐ Laurel ☐ Meridian ☐ Natchez ☐ Ocean Springs ☐ Oxford ☐ Pascagoula
☐ Pass Christian ☐ Philadelphia ☐ Port Gibson ☐ Starkville ☐ Tupelo ☐ Vicksburg
☐ West Point ☐ Yazoo City ☐ Not listed

Which cities in Missouri?

☐ Boonville ☐ Branson ☐ Cape Girardeau ☐ Carthage ☐ Chillicothe ☐ Clayton
☐ Columbia ☐ Excelsior Springs ☐ Ferguson ☐ Florissant ☐ Fulton ☐ Hannibal
☐ Independence ☐ Jefferson City ☐ Joplin ☐ Kansas City ☐ Kirksville ☐ Lamar
☐ Lebanon ☐ Lexington ☐ Maryville ☐ Mexico ☐ Monett ☐ Neosho ☐ New Madrid
☐ Rolla ☐ Saint Charles ☐ Saint Joseph ☐ Saint Louis ☐ Sainte Genevieve ☐ Salem
☐ Sedalia ☐ Springfield ☐ Warrensburg ☐ West Plains ☐ Not listed

Which cities in Montana?

☐ Anaconda ☐ Billings ☐ Bozeman ☐ Butte ☐ Dillon ☐ Fort Benton ☐ Glendive
☐ Great Falls ☐ Havre ☐ Helena ☐ Kalispell ☐ Lewistown ☐ Livingston ☐ Miles City
☐ Missoula ☐ Virginia City ☐ Not listed

Which cities in Nebraska?

☐ Beatrice ☐ Bellevue ☐ Boys Town ☐ Chadron ☐ Columbus ☐ Fremont ☐ Grand Island
☐ Hastings ☐ Kearney ☐ Lincoln ☐ McCook ☐ Minden ☐ Nebraska City ☐ Norfolk
☐ North Platte ☐ Omaha ☐ Plattsmouth ☐ Red Cloud ☐ Sidney ☐ Not listed

Which cities in Nevada?

☐ Boulder City ☐ Carson City ☐ Elko ☐ Ely ☐ Fallon ☐ Genoa ☐ Goldfield
☐ Henderson ☐ Las Vegas ☐ North Las Vegas ☐ Reno ☐ Sparks ☐ Virginia City
☐ Winnemucca ☐ Not listed

Which cities in New Hampshire?

☐ Berlin ☐ Claremont ☐ Concord ☐ Derry ☐ Dover ☐ Durham ☐ Exeter ☐ Franklin
☐ Hanover ☐ Hillsborough ☐ Keene ☐ Laconia ☐ Lebanon ☐ Manchester ☐ Nashua
☐ Peterborough ☐ Plymouth ☐ Portsmouth ☐ Rochester ☐ Salem ☐ Somersworth
☐ Not listed

Which cities in New Jersey?

☐ Asbury Park ☐ Atlantic City ☐ Bayonne ☐ Bloomfield ☐ Bordentown ☐ Bound Brook
☐ Bridgeton ☐ Burlington ☐ Caldwell ☐ Camden ☐ Cape May ☐ Clifton ☐ Cranford
☐ East Orange ☐ Edison ☐ Elizabeth ☐ Englewood ☐ Fort Lee ☐ Glassboro ☐ Hackensack
☐ Haddonfield ☐ Hoboken ☐ Irvington ☐ Jersey City ☐ Lakehurst ☐ Lakewood
☐ Long Beach ☐ Long Branch ☐ Madison ☐ Menlo Park ☐ Millburn ☐ Millville
☐ Montclair ☐ Morristown ☐ Mount Holly ☐ New Brunswick ☐ New Milford ☐ Newark
☐ Ocean City ☐ Orange ☐ Parsippany-Troy Hills ☐ Passaic ☐ Paterson ☐ Perth Amboy
☐ Plainfield ☐ Princeton ☐ Ridgewood ☐ Roselle ☐ Rutherford ☐ Salem ☐ Somerville
☐ South Orange Village ☐ Totowa ☐ Trenton ☐ Union ☐ Union City ☐ Vineland
☐ Wayne ☐ Weehawken ☐ West New York ☐ West Orange ☐ Willingboro ☐ Woodbridge
☐ Not listed

Which cities in New Mexico?

☐ Acoma ☐ Alamogordo ☐ Albuquerque ☐ Artesia ☐ Belen ☐ Carlsbad ☐ Clovis
☐ Deming ☐ Farmington ☐ Gallup ☐ Grants ☐ Hobbs ☐ Las Cruces ☐ Las Vegas
☐ Los Alamos ☐ Lovington ☐ Portales ☐ Raton ☐ Roswell ☐ Santa Fe ☐ Shiprock
☐ Silver City ☐ Socorro ☐ Taos ☐ Truth or Consequences ☐ Tucumcari ☐ Not listed

Which cities in New York?

☐ Albany ☐ Amsterdam ☐ Auburn ☐ Babylon ☐ Batavia ☐ Beacon ☐ Bedford
☐ Binghamton ☐ Bronx ☐ Brooklyn ☐ Buffalo ☐ Chautauqua ☐ Cheektowaga
☐ Clinton ☐ Cohoes ☐ Coney Island ☐ Cooperstown ☐ Corning ☐ Cortland ☐ Crown Point
☐ Dunkirk ☐ East Aurora ☐ East Hampton ☐ Eastchester ☐ Elmira ☐ Flushing
☐ Forest Hills ☐ Fredonia ☐ Garden City ☐ Geneva ☐ Glens Falls ☐ Gloversville
☐ Great Neck ☐ Hammondsport ☐ Harlem ☐ Hempstead ☐ Herkimer ☐ Hudson
☐ Huntington ☐ Hyde Park ☐ Ilion ☐ Ithaca ☐ Jamestown ☐ Johnstown ☐ Kingston
☐ Lackawanna ☐ Lake Placid ☐ Levittown ☐ Lockport ☐ Mamaroneck ☐ Manhattan
☐ Massena ☐ Middletown ☐ Mineola ☐ Mount Vernon ☐ New Paltz ☐ New Rochelle
☐ New Windsor ☐ New York City ☐ Newburgh ☐ Niagara Falls ☐ North Hempstead
☐ Nyack ☐ Ogdensburg ☐ Olean ☐ Oneida ☐ Oneonta ☐ Ossining ☐ Oswego
☐ Oyster Bay ☐ Palmyra ☐ Peekskill ☐ Plattsburgh ☐ Port Washington ☐ Potsdam
☐ Poughkeepsie ☐ Queens ☐ Rensselaer ☐ Rochester ☐ Rome ☐ Rotterdam
☐ Rye ☐ Sag Harbor ☐ Saranac Lake ☐ Saratoga Springs ☐ Scarsdale ☐ Schenectady
☐ Seneca Falls ☐ Southampton ☐ Staten Island ☐ Stony Brook ☐ Stony Point ☐ Syracuse
☐ Tarrytown ☐ Ticonderoga ☐ Tonawanda ☐ Troy ☐ Utica ☐ Watertown ☐ Watervliet
☐ Watkins Glen ☐ West Seneca ☐ White Plains ☐ Woodstock ☐ Yonkers ☐ Not listed

Which cities in North Carolina?

☐ Asheboro ☐ Asheville ☐ Bath ☐ Beaufort ☐ Boone ☐ Burlington ☐ Chapel Hill
☐ Charlotte ☐ Concord ☐ Durham ☐ Edenton ☐ Elizabeth City ☐ Fayetteville
☐ Gastonia ☐ Goldsboro ☐ Greensboro ☐ Greenville ☐ Halifax ☐ Henderson
☐ Hickory ☐ High Point ☐ Hillsborough ☐ Jacksonville ☐ Kinston ☐ Kitty Hawk
☐ Lumberton ☐ Morehead City ☐ Morganton ☐ Nags Head ☐ New Bern ☐ Pinehurst
☐ Raleigh ☐ Rocky Mount ☐ Salisbury ☐ Shelby ☐ Washington ☐ Wilmington
☐ Wilson ☐ Winston-Salem ☐ Not listed

Which cities in North Dakota?

☐ Bismarck ☐ Devils Lake ☐ Dickinson ☐ Fargo ☐ Grand Forks ☐ Jamestown
☐ Mandan ☐ Minot ☐ Rugby ☐ Valley City ☐ Wahpeton ☐ Williston ☐ Not listed

Which cities in Ohio?

☐ Akron ☐ Alliance ☐ Ashtabula ☐ Athens ☐ Barberton ☐ Bedford ☐ Bellefontaine
☐ Bowling Green ☐ Canton ☐ Chillicothe ☐ Cincinnati ☐ Cleveland ☐ Cleveland Heights
☐ Columbus ☐ Conneaut ☐ Cuyahoga Falls ☐ Dayton ☐ Defiance ☐ Delaware
☐ East Cleveland ☐ East Liverpool ☐ Elyria ☐ Euclid ☐ Findlay ☐ Gallipolis ☐ Greenville
☐ Hamilton ☐ Kent ☐ Kettering ☐ Lakewood ☐ Lancaster ☐ Lima ☐ Lorain
☐ Mansfield ☐ Marietta ☐ Marion ☐ Martins Ferry ☐ Massillon ☐ Mentor ☐ Middletown
☐ Milan ☐ Mount Vernon ☐ New Philadelphia ☐ Newark ☐ Niles ☐ North College Hill
☐ Norwalk ☐ Oberlin ☐ Painesville ☐ Parma ☐ Piqua ☐ Portsmouth ☐ Put-in-Bay
☐ Salem ☐ Sandusky ☐ Shaker Heights ☐ Springfield ☐ Steubenville ☐ Tiffin
☐ Toledo ☐ Urbana ☐ Warren ☐ Wooster ☐ Worthington ☐ Xenia ☐ Yellow Springs
☐ Youngstown ☐ Zanesville ☐ Not listed

Which cities in Oklahoma?

☐ Ada ☐ Altus ☐ Alva ☐ Anadarko ☐ Ardmore ☐ Bartlesville ☐ Bethany ☐ Chickasha
☐ Claremore ☐ Clinton ☐ Cushing ☐ Duncan ☐ Durant ☐ Edmond ☐ El Reno
☐ Elk City ☐ Enid ☐ Eufaula ☐ Frederick ☐ Guthrie ☐ Guymon ☐ Hobart ☐ Holdenville
☐ Hugo ☐ Lawton ☐ McAlester ☐ Miami ☐ Midwest City ☐ Moore ☐ Muskogee
☐ Norman ☐ Oklahoma City ☐ Okmulgee ☐ Pauls Valley ☐ Pawhuska ☐ Perry
☐ Ponca City ☐ Pryor ☐ Sallisaw ☐ Sand Springs ☐ Sapulpa ☐ Seminole ☐ Shawnee
☐ Stillwater ☐ Tahlequah ☐ The Village ☐ Tulsa ☐ Vinita ☐ Wewoka ☐ Woodward
☐ Not listed

Which cities in Oregon?

☐ Albany ☐ Ashland ☐ Astoria ☐ Baker City ☐ Beaverton ☐ Bend ☐ Brookings
☐ Burns ☐ Coos Bay ☐ Corvallis ☐ Eugene ☐ Grants Pass ☐ Hillsboro ☐ Hood River
☐ Jacksonville ☐ John Day ☐ Klamath Falls ☐ La Grande ☐ Lake Oswego ☐ Lakeview
☐ McMinnville ☐ Medford ☐ Newberg ☐ Newport ☐ Ontario ☐ Oregon City ☐ Pendleton
☐ Port Orford ☐ Portland ☐ Prineville ☐ Redmond ☐ Reedsport ☐ Roseburg
☐ Salem ☐ Seaside ☐ Springfield ☐ The Dalles ☐ Tillamook ☐ Not listed

Which cities in Pennsylvania?

☐ Abington ☐ Aliquippa ☐ Allentown ☐ Altoona ☐ Ambridge ☐ Bedford ☐ Bethlehem
☐ Bloomsburg ☐ Bradford ☐ Bristol ☐ Carbondale ☐ Carlisle ☐ Chambersburg
☐ Chester ☐ Columbia ☐ Easton ☐ Erie ☐ Franklin ☐ Germantown ☐ Gettysburg
☐ Greensburg ☐ Hanover ☐ Harmony ☐ Harrisburg ☐ Hazleton ☐ Hershey ☐ Homestead
☐ Honesdale ☐ Indiana ☐ Jeannette ☐ Jim Thorpe ☐ Johnstown ☐ Lancaster
☐ Lebanon ☐ Levittown ☐ Lewistown ☐ Lock Haven ☐ Lower Southampton ☐ McKeesport
☐ Meadville ☐ Middletown ☐ Monroeville ☐ Nanticoke ☐ New Castle ☐ New Hope
☐ New Kensington ☐ Norristown ☐ Oil City ☐ Philadelphia ☐ Phoenixville ☐ Pittsburgh
☐ Pottstown ☐ Pottsville ☐ Reading ☐ Scranton ☐ Shamokin ☐ Sharon ☐ State College
☐ Stroudsburg ☐ Sunbury ☐ Swarthmore ☐ Tamaqua ☐ Titusville ☐ Uniontown
☐ Warren ☐ Washington ☐ West Chester ☐ Wilkes-Barre ☐ Williamsport ☐ York
☐ Not listed

Which states in Rhode Island?

☐ Barrington ☐ Bristol ☐ Central Falls ☐ Cranston ☐ East Greenwich ☐ East Providence
☐ Kingston ☐ Middletown ☐ Narragansett ☐ Newport ☐ North Kingstown ☐ Pawtucket
☐ Portsmouth ☐ Providence ☐ South Kingstown ☐ Tiverton ☐ Warren ☐ Warwick
☐ Westerly ☐ Wickford ☐ Woonsocket ☐ Not listed

Which cities in South Carolina?

☐ Abbeville ☐ Aiken ☐ Anderson ☐ Beaufort ☐ Camden ☐ Charleston ☐ Columbia
☐ Darlington ☐ Florence ☐ Gaffney ☐ Georgetown ☐ Greenville ☐ Greenwood
☐ Hartsville ☐ Lancaster ☐ Mount Pleasant ☐ Myrtle Beach ☐ Orangeburg ☐ Rock Hill
☐ Spartanburg ☐ Sumter ☐ Union ☐ Not listed

Which cities in South Dakota?

☐ Aberdeen ☐ Belle Fourche ☐ Brookings ☐ Canton ☐ Custer ☐ De Smet ☐ Deadwood
☐ Hot Springs ☐ Huron ☐ Lead ☐ Madison ☐ Milbank ☐ Mitchell ☐ Mobridge
☐ Pierre ☐ Rapid City ☐ Sioux Falls ☐ Spearfish ☐ Sturgis ☐ Vermillion ☐ Watertown
☐ Yankton ☐ Not listed

Which cities in Tennessee?

☐ Alcoa ☐ Athens ☐ Chattanooga ☐ Clarksville ☐ Cleveland ☐ Columbia ☐ Cookeville
☐ Dayton ☐ Elizabethton ☐ Franklin ☐ Gallatin ☐ Gatlinburg ☐ Greeneville ☐ Jackson
☐ Johnson City ☐ Jonesborough ☐ Kingsport ☐ Knoxville ☐ Lebanon ☐ Maryville
☐ Memphis ☐ Morristown ☐ Murfreesboro ☐ Nashville ☐ Norris ☐ Oak Ridge
☐ Shelbyville ☐ Tullahoma ☐ Not listed

Which cities in Texas?

☐ Abilene ☐ Alpine ☐ Amarillo ☐ Arlington ☐ Austin ☐ Baytown ☐ Beaumont
☐ Big Spring ☐ Borger ☐ Brownsville ☐ Bryan ☐ Canyon ☐ Cleburne ☐ College Station
☐ Corpus Christi ☐ Crystal City ☐ Dallas ☐ Del Rio ☐ Denison ☐ Denton ☐ Eagle Pass
☐ Edinburg ☐ El Paso ☐ Fort Worth ☐ Freeport ☐ Galveston ☐ Garland ☐ Goliad
☐ Greenville ☐ Harlingen ☐ Houston ☐ Huntsville ☐ Irving ☐ Johnson City ☐ Kilgore
☐ Killeen ☐ Kingsville ☐ Laredo ☐ Longview ☐ Lubbock ☐ Lufkin ☐ Marshall
☐ McAllen ☐ McKinney ☐ Mesquite ☐ Midland ☐ Mission ☐ Nacogdoches ☐ New Braunfels
☐ Odessa ☐ Orange ☐ Pampa ☐ Paris ☐ Pasadena ☐ Pecos ☐ Pharr ☐ Plainview
☐ Plano ☐ Port Arthur ☐ Port Lavaca ☐ Richardson ☐ San Angelo ☐ San Antonio
☐ San Felipe ☐ San Marcos ☐ Sherman ☐ Sweetwater ☐ Temple ☐ Texarkana
☐ Texas City ☐ Tyler ☐ Uvalde ☐ Victoria ☐ Waco ☐ Weatherford ☐ Wichita Falls
☐ Ysleta ☐ Not listed

Which cities in Utah?

☐ Alta ☐ American Fork ☐ Bountiful ☐ Brigham City ☐ Cedar City ☐ Clearfield
☐ Delta ☐ Fillmore ☐ Green River ☐ Heber City ☐ Kanab ☐ Layton ☐ Lehi
☐ Logan ☐ Manti ☐ Moab ☐ Monticello ☐ Murray ☐ Nephi ☐ Ogden ☐ Orderville
☐ Orem ☐ Panguitch ☐ Park City ☐ Payson ☐ Price ☐ Provo ☐ Saint George
☐ Salt Lake City ☐ Spanish Fork ☐ Springville ☐ Tooele ☐ Vernal ☐ Not listed

Which cities in Vermont?

☐ Barre ☐ Bellows Falls ☐ Bennington ☐ Brattleboro ☐ Burlington ☐ Essex ☐ Manchester
☐ Middlebury ☐ Montpelier ☐ Newport ☐ Plymouth ☐ Rutland ☐ Saint Albans
☐ Saint Johnsbury ☐ Sharon ☐ Winooski ☐ Not listed

Which cities in Virginia

☐ Abingdon ☐ Alexandria ☐ Bristol ☐ Charlottesville ☐ Chesapeake ☐ Danville
☐ Fairfax ☐ Falls Church ☐ Fredericksburg ☐ Hampton ☐ Hanover ☐ Hopewell
☐ Lexington ☐ Lynchburg ☐ Manassas ☐ Martinsville ☐ New Market ☐ Newport News
☐ Norfolk ☐ Petersburg ☐ Portsmouth ☐ Reston ☐ Richmond ☐ Roanoke ☐ Staunton
☐ Suffolk ☐ Virginia Beach ☐ Waynesboro ☐ Williamsburg ☐ Winchester ☐ Not listed

Which cities in Washington?

☐ Aberdeen ☐ Anacortes ☐ Auburn ☐ Bellevue ☐ Bellingham ☐ Bremerton ☐ Centralia
☐ Coulee Dam ☐ Coupeville ☐ Ellensburg ☐ Ephrata ☐ Everett ☐ Hoquiam ☐ Kelso
☐ Kennewick ☐ Longview ☐ Moses Lake ☐ Oak Harbor ☐ Olympia ☐ Pasco ☐ Point Roberts
☐ Port Angeles ☐ Pullman ☐ Puyallup ☐ Redmond ☐ Renton ☐ Richland ☐ Seattle
☐ Spokane ☐ Tacoma ☐ Vancouver ☐ Walla Walla ☐ Wenatchee ☐ Yakima ☐ Not listed

Which cities in West Virginia?

☐ Bath ☐ Beckley ☐ Bluefield ☐ Buckhannon ☐ Charles Town ☐ Charleston ☐ Clarksburg
☐ Elkins ☐ Fairmont ☐ Grafton ☐ Harpers Ferry ☐ Hillsboro ☐ Hinton ☐ Huntington
☐ Keyser ☐ Lewisburg ☐ Logan ☐ Martinsburg ☐ Morgantown ☐ Moundsville
☐ New Martinsville ☐ Parkersburg ☐ Philippi ☐ Point Pleasant ☐ Princeton ☐ Romney
☐ Shepherdstown ☐ South Charleston ☐ Summersville ☐ Weirton ☐ Welch ☐ Wellsburg
☐ Weston ☐ Wheeling ☐ White Sulphur Springs ☐ Williamson ☐ Not listed

Which cities in Wisconsin?

☐ Appleton ☐ Ashland ☐ Baraboo ☐ Belmont ☐ Beloit ☐ Eau Claire ☐ Fond du Lac
☐ Green Bay ☐ Hayward ☐ Janesville ☐ Kenosha ☐ La Crosse ☐ Lake Geneva
☐ Madison ☐ Manitowoc ☐ Marinette ☐ Menasha ☐ Milwaukee ☐ Neenah ☐ New Glarus
☐ Oconto ☐ Oshkosh ☐ Peshtigo ☐ Portage ☐ Prairie du Chien ☐ Racine ☐ Rhinelander
☐ Ripon ☐ Sheboygan ☐ Spring Green ☐ Stevens Point ☐ Sturgeon Bay ☐ Superior
☐ Waukesha ☐ Wausau ☐ Wauwatosa ☐ West Allis ☐ West Bend ☐ Wisconsin Dells
☐ Not listed

Which cities in Wyoming?

☐ Buffalo ☐ Casper ☐ Cheyenne ☐ Cody ☐ Douglas ☐ Evanston ☐ Gillette
☐ Green River ☐ Jackson ☐ Lander ☐ Laramie ☐ Newcastle ☐ Powell ☐ Rawlins
☐ Riverton ☐ Rock Springs ☐ Sheridan ☐ Ten Sleep ☐ Thermopolis ☐ Torrington
☐ Worland ☐ Not listed

Which Non-US countries?

☐ Afghanistan ☐ Albania ☐ Algeria ☐ Andorra ☐ Angola ☐ Antigua and Barbuda
☐ Argentina ☐ Armenia ☐ Australia ☐ Austria ☐ Azerbaijan ☐ Bahamas ☐ Bahrain
☐ Bangladesh ☐ Barbados ☐ Belarus ☐ Belgium ☐ Belize ☐ Benin ☐ Bhutan
☐ Bolivia ☐ Bosnia and Herzegovina ☐ Botswana ☐ Brazil ☐ Brunei ☐ Bulgaria
☐ Burkina Faso ☐ Burundi ☐ Cabo Verde ☐ Cambodia ☐ Cameroon ☐ Canada
☐ Central African Republic (CAR) ☐ Chad ☐ Chile ☐ China ☐ Colombia ☐ Comoros
☐ Democratic Republic of the Congo ☐ Republic of the Congo ☐ Costa Rica ☐ Cote d'Ivoire
☐ Croatia ☐ Cuba ☐ Cyprus ☐ Czech Republic ☐ Denmark ☐ Djibouti ☐ Dominica
☐ Dominican Republic ☐ Ecuador ☐ Egypt ☐ El Salvador ☐ Equatorial Guinea ☐ Eritrea
☐ Estonia ☐ Eswatini (formerly Swaziland) ☐ Ethiopia ☐ Fiji ☐ Finland ☐ France
☐ Gabon ☐ Gambia ☐ Georgia ☐ Germany ☐ Ghana ☐ Greece ☐ Grenada
☐ Guatemala ☐ Guinea ☐ Guinea-Bissau ☐ Guyana ☐ Haiti ☐ Honduras ☐ Hungary
☐ Iceland ☐ India ☐ Indonesia ☐ Iran ☐ Iraq ☐ Ireland ☐ Israel ☐ Italy ☐ Jamaica
☐ Japan ☐ Jordan ☐ Kazakhstan ☐ Kenya ☐ Kiribati ☐ Kosovo ☐ Kuwait ☐ Kyrgyzstan
☐ Laos ☐ Latvia ☐ Lebanon ☐ Lesotho ☐ Liberia ☐ Libya ☐ Liechtenstein
☐ Lithuania ☐ Luxembourg ☐ Macedonia (FYROM) ☐ Madagascar ☐ Malawi ☐ Malaysia
☐ Maldives ☐ Mali ☐ Malta ☐ Marshall Islands ☐ Mauritania ☐ Mauritius ☐ Mexico
☐ Micronesia ☐ Moldova ☐ Monaco ☐ Mongolia ☐ Montenegro ☐ Morocco ☐ Mozambique
☐ Myanmar (formerly Burma) ☐ Namibia ☐ Nauru ☐ Nepal ☐ Netherlands ☐ New Zealand
☐ Nicaragua ☐ Niger ☐ Nigeria ☐ North Korea ☐ Norway ☐ Oman ☐ Pakistan
☐ Palau ☐ Palestine ☐ Panama ☐ Papua New Guinea ☐ Paraguay ☐ Peru ☐ Philippines
☐ Poland ☐ Portugal ☐ Qatar ☐ Romania ☐ Russia ☐ Rwanda ☐ Saint Kitts and Nevis
☐ Saint Lucia ☐ Saint Vincent and the Grenadines ☐ Samoa ☐ San Marino ☐ Sao Tome and Principe
☐ Saudi Arabia ☐ Senegal ☐ Serbia ☐ Seychelles ☐ Sierra Leone ☐ Singapore
☐ Slovakia ☐ Slovenia ☐ Solomon Islands ☐ Somalia ☐ South Africa ☐ South Korea
☐ South Sudan ☐ Spain ☐ Sri Lanka ☐ Sudan ☐ Suriname ☐ Swaziland (renamed to Eswatini)
☐ Sweden ☐ Switzerland ☐ Syria ☐ Taiwan ☐ Tajikistan ☐ Tanzania ☐ Thailand
☐ Timor-Leste ☐ Togo ☐ Tonga ☐ Trinidad and Tobago ☐ Tunisia ☐ Turkey ☐ Turkmenistan
☐ Tuvalu ☐ Uganda ☐ Ukraine ☐ United Arab Emirates (UAE) ☐ United Kingdom (UK)
☐ Uruguay ☐ Uzbekistan ☐ Vanuatu ☐ Vatican City (Holy See) ☐ Venezuela ☐ Vietnam
☐ Yemen ☐ Zambia ☐ Zimbabwe ☐ Scottland

Which Cities? (if applicable)

____

Outcome Type

☐ Incidence
☐ Mortality
☐ Other (Notes)

Other type of outcome

____

Analysis type

☐ Case Control/ Odds Ratio
☐ Standardized Incidence Ratio
☐ Standardized Mortality Ratio
☐ Proportional Mortality Ratio
☐ Frequency
☐ Other (Notes)

Other type of analysis

____

Total sample size

____

Total Person-Years

____

SIR n=

____

Source of occupation designation

☐ Employment records
☐ Certification records
☐ Cancer registry records
☐ Death certificate records
☐ Other (Notes)
☐ Not Reported

Other (Notes)

____

DX coding system

☐ ICD-6 ☐ ICD-7 ☐ ICD-8 ☐ ICD-9 ☐ ICD-10 ☐ Other (Notes) ☐ Not specified

ICD-10 code

____
(Cause of death, ICD-10 Code)

Please provide the page number of the article with the codes on it.

Other (Notes)

Age

☐ At cohort start date
☐ At cohort end date
☐ At death
☐ At diagnosis
☐ At hire
☐ Not reported

Age at cohort start date: Mean

____

Age at cohort start date : Standard Deviation

____
(If variance is given please convert to standard deviation)

Age at cohort start date: Min

____

Age at cohort start date: Max

____

Age at cohort start
Frequencies and percentages

____

Age at cohort end date: Mean

____

Age at cohort end date : Standard Deviation

____
(If variance is given please convert to standard deviation)

Age at cohort end date: Min

____

Age at cohort end date: Max

____

Age at cohort end
Frequencies and percentages

____

Age at death: Mean

____

Age at death : Standard Deviation

____
(If variance is given please convert to standard deviation)

Age at death: Min

____

Age at death: Max

____

Age at death
Frequencies and percentages

____

Age at dx: Mean

____

Age at dx : Standard Deviation

____
(If variance is given please convert to standard deviation)

Age at dx: Min

____

Age at dx: Max

____

Age at dx<br>Frequencies and percentages	______________________
Age at hire: Mean	______________________
Age at hire: SD	______________________<br>(if variance is reported please convert it to standard deviation and type here)
Age at hire: Min	______________________
Age at hire: Max	______________________
Age at hire<br>Frequencies and percentages	______________________
Is duration of employment in years collected categorically or continuously?	◯ Categorically<br>◯ Continuous
Employment in years<br>Mean:	______________________
Employment in years<br>SD:	______________________<br>((if variance is reported please convert it to standard deviation and type here))
Employment in years<br>Min:	______________________
Employment in years<br>Max:	______________________
Type in the categories best fitting for employment in years.	______________________
Employment status	◯ Part-time<br>◯ Full-time<br>◯ Other (Notes)<br>◯ Not reported
Other employment status	______________________

Type of incident attended	☐ All fires<br>☐ Landscape fires<br>☐ Vehicle fires<br>☐ Structural<br>☐ Other (Notes)<br>☐ Not specified
Other incident type	__________________________________
Era of first employment	◯ Continuous<br>◯ Categorical<br>(Era of employment: When they were first certified or first started working as a firefighter so that we can accurately document how long they have been exposed.)
Mean year of first employment	___________________________
SD year of first employment	___________________________<br>((if variance is reported please convert it to standard deviation and type here))
Minimum year of first employment	___________________________
Maximum year of first employment	___________________________
Type in the categories for Era of first employment	__________________________________
Gender	☐ Male<br>☐ Female<br>☐ Other (Please specify)<br>☐ Not reported
Other	__________________________________
Male n=	___________________________
Male %=	___________________________
Female n=	___________________________
Female %=	___________________________

Other n=

____

Other %=

____

Race/ Ethnicity

☐ White ☐ Black ☐ Asian ☐ Other ☐ Unknown ☐ Not reported ☐ Notes ☐ Hispanic

White n=

____

White %=

____

Black n=

____

Black %=

____

Asian n=

____

Asian %=

____

Other

____
(please list the other race)

other n=

____

other %=

____

Unknown n=

____

Unknown %=

____

Notes

____

Hispanic n=

____

Hispanic %=

____

Mortality follow-up

Alive n= ______________________

Alive %= ______________________

Dead n= ______________________

Dead %= ______________________

Person years of follow up
Mean= ______________________

Person years of follow up
SD= ______________________

Person years of follow up
Min= ______________________

Person years of follow up
Max= ______________________

Average length of follow up in years
Mean= ______________________

Length of follow-up in years
SD= ______________________

Deaths notified by agencies
n= ______________________

Deaths notified by agencies
%= ______________________

Deaths from linkage
n= ______________________

Deaths from linkage
%= ______________________

Age at death, if deceased
Mean= ______________________

Age at death, if deceased
SD= ______________________

Person years of follow up
Mean= ______________________

Person years of follow up
SD= ______________________

Person years of follow up
Min= ______________________________

Person years of follow up
Max= ______________________________

First Primary Analysis
◯ Yes
◯ No
◯ Not applicable
(First primaries are the first diagnosed cancer for a particular person and secondary primaries are any subsequent cancers diagnosed in the same person, but not including metastases.)

Multiple Primary analysis
◯ Yes
◯ No
◯ Not applicable

Smoking Information

Smoking Status
◯ Never smoker
◯ Former smoker
◯ Current smoker
◯ Ever smoker
◯ Not reported

Are the cigarette pack years collected continuously or categorically?
◯ Continuously
◯ Categorically
◯ Not reported

Cigarette pack years
Mean= ______________________________

Cigarette pack years
SD= ______________________________

Cigarette pack years
Min= ______________________________

Cigarette pack years
Max= ______________________________

Cigarette pack years
◯ < 10 pack years
◯ 10-20 pack years
◯ 21+ pack years
