# Supplementary material for: Non-cancer mortality among firefighters: a meta-analytic review of heart disease, stroke, respiratory disease, liver disease, accidents, and suicide
Source: Front Public Health. 2026 Feb 26;14:1714033. doi: 10.3389/fpubh.2026.1714033 (PMC12979482; doi:10.3389/fpubh.2026.1714033)
Supplement: Supplementary file 3 [file Data_Sheet_3.DOCX]

**Literature Search for Incidence and Mortality among Firefighters**

**PUBMED:**

**Search terms:**

(“mortality” OR "cause of death" OR "cause specific mortality" OR “all cause mortality”) AND

(firealarm[All Fields] OR firebomb [All Fields] OR firebombed[All Fields] OR firebombing[All Fields] OR firebombings[All Fields] OR firebreak[All Fields] OR firebreaks[All Fields] OR fireburn[All Fields] OR firebush[All Fields] OR firecenter[All Fields] OR firechief[All Fields] OR firecode[All Fields] OR firedepartment[All Fields] OR “fire department”[All Fields] OR firefighter[All Fields] OR firefighters[All Fields] OR firefighters’[All Fields] OR firefighing[All Fields] OR firefight[All Fields] OR “fire fighter”[All Fields] OR firefighter’[All Fields] OR firefighter’s[All Fields] OR firefighters[All Fields] OR firefighters’[All Fields] OR firefighting[All Fields] OR firefights[All Fields] OR fireground[All Fields] OR firegrounds[All Fields] OR fireguard[All Fields] OR fireguards[All Fields] OR fireguide [All Fields] OR firehouse[All Fields] OR firehouses[All Fields] OR fireman[All Fields] OR fireman**’**[All Fields] OR fireman’s[All Fields] OR firemen[All Fields] OR firemen’s[All Fields] OR fireperson[All Fields] OR firepersons[All Fields] OR firerangers[All Fields] OR fireservice[All Fields] OR “fire service” [All Fields] OR “fire inspector” [All Fields] OR “fire inspectors” [All Fields] OR “fire rescue” [All Fields] OR “firerescue” [All Fields] OR paramedic [All Fields] OR paramedics [All Fields] OR EMT [All Fields] OR “Emergency Medical Technician” [All Fields] OR “Wildland” [All Fields])

**Search Limits:**

Species: Human

Language: English

Age:

Adult: 19+ years

Publication date: January 1, 1978 - December 31, 2025

**EMBASE:**

Calder.med.miami.edu → Embase→ Advanced search

**Search terms:**

('mortality'/exp OR mortality:ab,ti OR 'cause of death'/exp)

AND

(

'firefighter'/exp

OR firefighter:ab,ti

OR "fire fighter":ab,ti

OR firefighters:ab,ti

OR paramedic:ab,ti

OR paramedics:ab,ti

OR 'emergency medical technician':ab,ti

OR 'first responder':ab,ti

)

AND [english]/lim

AND [humans]/lim

AND [adult]/lim

AND [1978-2023]/py

**Search Limits:**

Species: Human

Language: English

Fields: abstract

Age:

Adult (18-64 years)

Aged (65+ years)

Publication date: January 1, 1978 - December 31, 2025

**Web of Science:**

Calder.med.miami.edu → Web of Science→ Advanced search

**Search terms:**

(Mortality OR cause of death OR “cause specific mortality”) (Abstract) and (fire inspector OR fire inspectors OR fire rescue OR fire-rescue OR firefighter OR firefighters OR "fire fighter" OR "fire fighters" OR paramedic OR paramedics OR emergency medical technician OR "first responder") (Abstract)

**Search Limits:**

Language: English

Fields: abstract

Publication date: January 1, 1978 - December 31, 2025

**EBSCO for PsychInfo:**

Calder.med.miami.edu → PsychInfo→ Advanced search

**Search terms:**

(Mortality OR cause of death OR “cause specific mortality”) (Title or Abstract) and (fire inspector OR fire inspectors OR fire rescue OR fire-rescue OR firefighter OR firefighters OR "fire fighter" OR "fire fighters" OR paramedic OR paramedics OR emergency medical technician OR "first responder") (Title or Abstract)

**Search Limits:**

Language: English

Fields: title or abstract

Age:

Adulthood (18+)

Middle Age (40-64 years)

Aged (65+ years & older)

Publication date: January 1, 1978 - December 31, 2025

**EBSCO for Medline:**

Calder.med.miami.edu → Medline→ Advanced search

**Search terms:**

(Mortality OR cause of death OR “cause specific mortality”) (Title or Abstract) and (fire inspector OR fire inspectors OR fire rescue OR fire-rescue OR firefighter OR firefighters OR "fire fighter" OR "fire fighters" OR paramedic OR paramedics OR emergency medical technician OR "first responder") (Title or Abstract)

**Search Limits:**

Language: English

Fields: title or abstract

Age:

All Adult (19+ years)

Publication date:

Publication date: January 1, 1978 - December 31, 2025

**SCOPUS:**

Calder.med.miami.edu → SCOPUS→ Advanced search

**Search terms:**

TITLE-ABS-KEY(mortality OR "cause of death" OR "cause specific mortality")AND TITLE-ABS-KEY(firefighter OR paramedic OR "emergency medical technician" OR "first responder") AND PUBYEAR > 1977 AND PUBYEAR < 2025 AND ( LIMIT-TO ( LANGUAGE,"English" ) ) AND ( LIMIT-TO ( EXACTKEYWORD,"Adult" ) )

**Search Limits:**

Language: English

Fields: title or abstract

Age:

Adult

Publication date: January 1, 1978 - December 31, 2025

**ProQuest:**

Calder.med.miami.edu → ProQuest→ Advanced search

**Search terms:**

abstract((mortality OR "cause of death" OR "cause specific mortality")) AND title((mortality OR "cause of death" OR "cause specific mortality")) AND abstract((fire inspector OR fire inspectors OR fire rescue OR fire-rescue OR firefighter OR firefighters OR "fire fighter" OR "fire fighters" OR paramedic OR paramedics OR emergency medical technician OR "first responder")) AND title((fire inspector OR fire inspectors OR fire rescue OR fire-rescue OR firefighter OR firefighters OR "fire fighter" OR "fire fighters" OR paramedic OR paramedics OR emergency medical technician OR "first responder"))

**Search Limits:**

Subject: humans

Language: English

Fields: title or abstract

Publication date: January 1, 1978 - December 31, 2025

**EBSCO for ERIC:**

Calder.med.miami.edu → ERIC→ Advanced search

**Search terms:**

(Mortality OR cause of death OR “cause specific mortality”) & (fire inspector OR fire inspectors OR fire rescue OR fire-rescue OR firefighter OR firefighters OR "fire fighter" OR "fire fighters" OR paramedic OR paramedics OR emergency medical technician OR "first responder")

**Search Limits:**

Subject: humans

Language: English

Fields: title or abstract

Publication date: January 1, 1978 - December 31, 2025
